# Transcriptomic profiling reveals histone acetylation-regulated genes involved in somatic embryogenesis in *Arabidopsis thaliana*

**DOI:** 10.1186/s12864-024-10623-5

**Published:** 2024-08-15

**Authors:** Barbara Wójcikowska, Karolina Chwiałkowska, Katarzyna Nowak, Sylvie Citerne, Joanna Morończyk, Anna Maria Wójcik, Agnieszka Kiwior-Wesołowska, Jacek Francikowski, Mirosław Kwaśniewski, Małgorzata Danuta Gaj

**Affiliations:** 1 Faculty of Natural Sciences, Institute of Biology, Biotechnology and Environmental Protection, University of Silesia in Katowice, Katowice, Poland; 2 Centre for Bioinformatics and Data Analysis, Medical University of Bialystok, Bialystok, Poland; 3 Université Paris-Saclay, INRAE, AgroParisTech, Institut Jean-Pierre Bourgin (IJPB), Versailles, 78000 France

**Keywords:** Auxin, Organ-polarity genes, RNA-seq, Somatic embryogenesis, Trichostatin A

## Abstract

**Background:**

Somatic embryogenesis (SE) exemplifies the unique developmental plasticity of plant cells. The regulatory processes, including epigenetic modifications controlling embryogenic reprogramming of cell transcriptome, have just started to be revealed.

**Results:**

To identify the genes of histone acetylation-regulated expression in SE, we analyzed global transcriptomes of Arabidopsis explants undergoing embryogenic induction in response to treatment with histone deacetylase inhibitor, trichostatin A (TSA). The TSA-induced and auxin (2,4-dichlorophenoxyacetic acid; 2,4-D)-induced transcriptomes were compared. RNA-seq results revealed the similarities of the TSA- and auxin-induced transcriptomic responses that involve extensive deregulation, mostly repression, of the majority of genes. Within the differentially expressed genes (DEGs), we identified the master regulators (transcription factors - TFs) of SE, genes involved in biosynthesis, signaling, and polar transport of auxin and NITRILASE-encoding genes of the function in indole-3-acetic acid (IAA) biosynthesis. TSA-upregulated *TF* genes of essential functions in auxin-induced SE, included *LEC1/LEC2*, *FUS3*, *AGL15*, *MYB118*, *PHB*, *PHV*, *PLTs*, and *WUS/WOXs*. The TSA-induced transcriptome revealed also extensive upregulation of stress-related genes, including those related to stress hormone biosynthesis. In line with transcriptomic data, TSA-induced explants accumulated salicylic acid (SA) and abscisic acid (ABA), suggesting the role of histone acetylation (Hac) in regulating stress hormone-related responses during SE induction. Since mostly the adaxial side of cotyledon explant contributes to SE induction, we also identified organ polarity-related genes responding to TSA treatment, including *AIL7/PLT7*, *RGE1*, *LBD18*, *40*, *HB32*, *CBF1*, and *ULT2*. Analysis of the relevant mutants supported the role of polarity-related genes in SE induction.

**Conclusion:**

The study results provide a step forward in deciphering the epigenetic network controlling embryogenic transition in somatic cells of plants.

**Supplementary Information:**

The online version contains supplementary material available at 10.1186/s12864-024-10623-5.

## Background

Plant somatic cells possess a unique capacity to develop into somatic embryos following somatic embryogenesis (SE), a process rarely observed *in planta* [1] but successfully induced in vitro in different plant species [2]. SE exemplifies the developmental plasticity of plant cells which involves the capacity for de- and re-differentiation into specific cell types/organs/somatic embryos in response to induction signals such as stress factors and hormone treatment [3]. In biotechnology, plant regeneration via in vitro-induced SE is widely applied in clonal propagation and genetic transformation of various plant species [4, 5]. SE provides also a valuable experimental system in studies of exo- and endogenous factors determining developmental plasticity and embryogenic transition in plant somatic cells. In particular, studies on SE induction in a model plant of Arabidopsis have contributed significantly to understanding the embryogenic reprogramming of plant somatic cells at the molecular level [6, 7]. Queries on the molecular factors controlling SE induction that have been addressed in Arabidopsis revealed a complex regulatory network in which the transcription factors (TFs), microRNAs (miRNAs), and epigenetic modifications interact to erase the existing cell identity and switch on the embryogenic pathway of development in already differentiated cells [8–10]. The *TFs* of essential role in SE induction include *LEAFY COTYLEDON1* (*LEC1*), *LEAFY COTYLEDON2* (*LEC2*), *ABSCISIC ACID INSENSITIVE3* (*ABI3*), *FUSCA3* (*FUS3*), *AGAMOUS-LIKE15* (*AGL15*), *WUSCHEL* (*WUS*), *WUSCHEL RELATED HOMEOBOX* genes (*WOXs*), and members of the *PLETHORA* (*PLT*) gene family including *BABY BOOM* (*BBM*) [6, 10, 11].

Deregulation of genes in embryogenic transition involves changes in chromatin accessibility controlled by epigenetic modifications [12, 13]. The role of DNA methylation in regulating genes, including *TFs*, in SE induction was demonstrated [14–17]. The interplay between DNA and histone methylation in controlling the in vitro-induced developmental processes, including SE induction, was reported [18–20]. In particular, the role of the gene-repressive complexes, PRC1 and PRC2, in regulating the SE-essential genes was evidenced [21–23].

The methylation of DNA and histones cooperates with histone acetylation to control genes in plant development [24, 25]. An acetylated state of histones results from the opposing activities of histone acetyltransferases (HATs) and histone deacetylases (HDACs) [26]. HATs neutralize the positively charged amino group of specific lysine residues rendering DNA more accessible to DNA-binding regulatory proteins, including TFs [27]. Opposingly to HATs, HDACs remove the acetyl groups from histones and reduce DNA accessibility and binding of the regulatory proteins to DNA [28].

An experimental approach in studies on the role of histone acetylation (Hac) in gene regulation relies on the chemical inhibition of HAT and HDAC activity [29–31]. Relevantly, trichostatin A (TSA), an antifungal antibiotic isolated from *Streptomyces hygroscopicus* of inhibitory effect on zinc-dependent HDACs (RPD3/HDA1 and HD2-type families), has been recommended to affect Hac [32]. TSA was documented to increase H3 and H4 acetylation at a global and gene-specific level, and the TSA-hyperacetylated epigenetic marks involve H3K9/K14 and H4K5 [33, 34].

In line with promoting Hac, TSA treatment was evidenced to increase chromatin accessibility in animals [35] and plants [36]. Relevantly its chromatin de-condensation activity, TSA has been recommended to improve the efficiency of animal cloning [37–39]. TSA treatment was recommended in plants to improve androgenesis and double haploid production in anther, and microspore cultures [40–43]. The TSA treatment of explants improved SE induction in different plants, including grapevine [44], conifers [45, 46], and coffee [47].

These reports demonstrated that TSA might substitute for the requirement of auxin treatment essential for SE induction in Arabidopsis and suggested the role of Hac in the regulation of genes controlling plant cell reprogramming. In support of this postulate, global changes in Hac level in microspore cultures of rape [48] and Arabidopsis explants [49] were indicated. Indirect pieces of evidence on the Hac contribution to the embryogenic reprogramming of plant somatic cells involve differential expression of *HAT* and *HDAC* genes in SE and the altered embryogenic response of the relevant *hat* and *hdac* mutants [40, 49, 50].

The mechanism by which Hac, possibly in cooperation with auxin, might regulate genes in SE induction remains mostly elusive. Most reports on the Hac role in controlling specific genes in SE provide indirect evidence [51–53]. Only recently, the direct impact of Hac on transcriptional regulation of genes in SE, including *LEC1*, *LEC2*, *FUS3*, *MYB118* [49], *AGL15* [54], and *WUS* [55] has been demonstrated.

RNA-seq analysis of SE transcriptome in response to TSA treatment might be a helpful analytical approach to identify the Hac-targeted genes controlling embryogenic transition in plant somatic cells. RNA-seq was successfully applied to identifying genes engaged in plant development and subjected to epigenetic regulation [23, 56–58].

Here, to gain insights into the Hac-regulated genes during the embryogenic transition in plant somatic cells, we analyzed transcriptomes of Arabidopsis explants treated with TSA and synthetic auxin (2,4-dichlorophenoxyacetic acid; 2,4-D). Metadata analysis of RNA-seq results at global and gene levels aimed at comparing the TSA- and auxin-induced transcriptomic changes and indicating candidate genes of Hac-regulated expression in SE induction. The candidate genes included the *TF* genes of the master regulatory function in SE, auxin- and stress-related genes, and the genes of reported functions in the specification of organ polarity. The identified TSA-deregulated candidates provide a unique set of data for studies on histone acetylation-mediated regulation of SE induction.

## Methods

### Plant material

The Columbia (Col-0) genotype of *Arabidopsis thaliana* (L.) Heynh. Col-0, insertional and chemical-induced mutant seeds were supplied by NASC (The Nottingham Arabidopsis Stock Centre) (Additional file 1).

### In vitro culture of explants and SE induction

Immature zygotic embryos (IZEs) of Arabidopsis at the cotyledonary stage of development were used as explants for the in vitro culture. The explants were isolated from siliques and cultured following the standard protocol for SE [59] induction in Arabidopsis. A basal E0 medium contained 3.2 g L^− 1^ of B5 micro and macro-elements (Duchefa Biochemie; #G0210) [60], 20 g L^− 1^ sucrose and 8 g L^− l^ agar, pH 5.8. A standard medium for SE induction (EA) contained 5.0 µM of 2,4-D (Sigma-Aldrich #D7299), [59]. In addition, E0 medium supplemented with TSA (Sigma Aldrich; #T1952) at a concentration of 1.0 µM was applied for SE induction [50]. Two parameters, i.e., SE efficiency, calculated as the frequency of the explants that produced somatic embryos, and SE productivity, calculated as the average number of somatic embryos per explant, were used to analyze the SE capacity of studied lines. Thirty explants in at least three replicates were evaluated for each line.

### Plant growth and in vitro culture conditions

Seed-derived plants were grown in Jiffy*-*7 (Jiffy, Norway) pots at 22 °C under a 16 h photoperiod of 100 µM m^− 2^ s^− 1^ white, fluorescent light. Plant materials that were grown in sterile conditions were kept at 23 °C under a 16 h photoperiod of 40 µM m^− 2^ s^− 1^ white, fluorescent light [50].

### RNA isolation, library preparation, and RNA-seq

An RNAqueous^®^ Total RNA Isolation Kit (ThermoScientific) was used to isolate total RNA from the IZE explants induced on different media (E0, EA, ET) for 5 and 10 days. The freshly isolated tissues were wiped in frozen mortars. Depending on the age of the culture, from 250 (5th day) to 100 (10th day) explants were used for RNA isolation per repetition. RNA isolation, library preparation, and sequencing were produced in three biological replicates. The concentration and purity of RNA samples were evaluated with an ND-1000 spectrophotometer (NanoDrop Technologies) [50]. The integrity of the RNA was determined using an Agilent 2100 Bioanalyzer and Agilent RNA 6000 Nano chips (Agilent Technologies, Santa Clara, USA). RNA samples were treated with RNase-Free DNase and then purified using the Acid-Phenol: Chloroform with ammonium acetate method (ThermoScientific). The sequencing libraries were prepared using Illumina ScriptSeq™ Complete Kit (Plant; Illumina, San Diego, USA) following the manufacturer’s protocol. Two micrograms of total RNA per sample were used as an input. Briefly, library prep involved the subsequent steps: ribosomal RNA removal with Ribo-Zero rRNA Removal Reagents (Plant Leaf; Illumina, San Diego, USA) followed by ethanol precipitation of the rRNA-depleted sample, RNA fragmentation, cDNA synthesis, RNA removal, terminal tagging of cDNA followed by bead cleanup, PCR amplification using Illumina indexes and final bead purification. The quality of the prepared Illumina libraries was analyzed using Agilent Bioanalyzer with the Agilent High Sensitivity DNA Kit (Agilent Technologies, Santa Clara, USA), and the quantities were estimated using a Qubit Fluorometer (Thermo Fisher Scientific, Waltham, USA). For cluster generation, the libraries were pooled with equimolar concentration and sequenced using the Illumina HiSeq 4000 system (Illumina, San Diego, USA) in 2 × 76 cycles paired-end (PE) mode with 6 barcoded samples per lane [49].

### RNA-seq data analysis

Sequencing data was processed to obtain fastq files with the bcl2fastq pipeline (Illumina, San Diego, USA) including demultiplexing and adapter trimming steps as previously described [49]. The quality of raw sequencing reads was evaluated with FastQC software [61], and all reports were compared with the MultiQC tool [62]. As all reads were high quality, they were only soft-trimmed and filtered with Sickle [63]. Then SortMeRNA was used to filter out left-over fragments originating from rRNAs [64]. The quality of cleaned reads was assessed again using FastQC [61] and MultiQC [62]. Cleaned reads were mapped to *Arabidopsis thaliana* genome assembly GCA_000001735.1 (TAIR10) using splice-aware aligner STAR [65], with mapping parameters adjusted to *A. thaliana* genome characteristics, basic two-pass mode, and allowing for 5% of mismatches to reference genome. Unique counts per gene were calculated with an in-bulit option in STAR and used for data visualization and further differential gene expression analysis. The quality of mapping was assessed with the SAMStat package [66], as well as Qualimap [67]. Sequence alignment files were indexed using SAMtools [68], and mapped reads were visually inspected by applying the Integrative Genomics Viewer (IGV; [69]. All further computational and graphical analyses were performed in the *R* environment. Samples size factors were estimated using the median ratio method and counts were normalized with the DESeq2 algorithm [70]. For data inspection and visualization, counts have regularized log-transformed (rlog) to get log2-scaled data that is approximately homoscedastic and normalized concerning library size. Hierarchical clustering of samples was performed based on distance expressed as an inverse of Pearson’s correlation and applying Ward D2 linkage algorithm. Principal component analysis was performed in the *R* environment and ‘prcomp’ function. Heatmaps were visualized with the ‘pheatmap’ package and ‘heatmap.2’ function from ‘gplots’ library.

### Statistical analysis

Differential expression analysis was performed with DESeq2 software [70] assuming negative binomial distribution, and applying a general linearized model with beta prior shrinkage. Wald’s exact test was used to call differentially expressed genes (DEGs) under |log2FC| ≥ 1 along with P-value adjustment for multiple comparisons with Benjamini-Hochberg False Discovery Rate (FDR) correction [71] under α = 0.05. Enrichment of gene ontology (GO) terms for DEGs were performed using Metascape (http://metascape.org/gp/index.html#/main/step1) [72] or ShinyGO v.0.80 (http://bioinformatics.sdstate.edu/go/) [73]. In tryptophan, hormone and RT-qPCR studies a two-way ANOVA (*p* < 0.05) followed by Tukey’s honestly significant difference test (Tukey HSD-test) (*p* < 0.05) (Statistica 12.0) was applied to calculate significant differences at least *p* < 0.05 between the compared samples. In SE capacity analysis the Student t-test was used to calculate any significant differences (at *p* < 0.05) between the combinations that were being compared.

### Evaluation of hormone content

The concentration of phytohormones, abscisic acid (ABA), salicylic acid (SA), jasmonic acid (JA), indole-3-acetic acid (IAA), was evaluated in the 5 d, 10 d cultured explants of Col-0 on E0, ET and EA (Additional files 2 and 3). For analysis, fresh tissue was immediately frozen in liquid nitrogen and stored at -80 °C. Depending on the age of the culture, from 500 (5th day) to 100 (10th day) explants per repetition were collected to analyze hormone content. Analysis was performed on three biological replicates. For each sample, 30 mg of fresh powder were extracted with 0.8 mL of acetone/water/acetic acid (80/19/1 v: v:v). Abscisic acid, salicylic acid, jasmonic acid, indole-3-acetic acid stable labelled isotopes used as internal standards were prepared as described in [74]. 1 ng of each standard was added to the sample. The extract was vigorously shaken for 1 min, sonicated for 1 min at 25 Hz, shaken for 10 min at 10 °C in a Thermomixer (Eppendorf^®^, and then centrifuged (8,000 g, 10 °C, 10 min.). The supernatants were collected, and the pellets were re-extracted twice with 0.4 mL of the same extraction solution, then vigorously shaken (1 min) and sonicated (1 min; 25 Hz). After the centrifugations, the three supernatants were pooled and dried (Final Volume 1.6 mL). Each dry extract was dissolved in 100 µL of acetonitrile/water (50/50 v/v), filtered, and analyzed using a Waters Acquity ultra performance liquid chromatograph coupled to a Waters Xevo Triple quadrupole mass spectrometer TQS (UPLC-ESI-MS/MS). The compounds were separated on a reverse-phase column (Uptisphere C18 UP3HDO, 100*2.1 mm*3µm particle size; Interchim, France) using a flow rate of 0.4 mL min^− 1^ and a binary gradient: (A) acetic acid 0.1% in water (v/v) and (B) acetonitrile with 0.1% acetic acid, the column temperature was 40 °C, for abscisic acid, salicylic acid, jasmonic acid, and indole-3-acetic acid we used the following binary gradient (time, % A): (0 min., 98%), (3 min., 70%), (7.5 min., 50%), (8.5 min., 5%), (9.6 min., 0%), (13.2 min., 98%), (15.7 min., 98%). Mass spectrometry was conducted in electrospray and Multiple Reaction Monitoring scanning mode (MRM mode), in positive ion mode for the indole-3-acetic acid, and in negative ion mode for the other hormones. Relevant instrumental parameters were set as follows: capillary 1.5 kV (negative mode), source block and desolvation gas temperatures 130 °C and 500 °C, respectively. Nitrogen was used to assist the cone and desolvation (150 L h^− 1^ and 800 L h^− 1^, respectively), argon was used as the collision gas at a flow of 0.18 mL min^− 1^.

### Evaluation of tryptophan (Trp) content

Thin-layer chromatography (TLC) was used as one of the most fundamental methods for aminoacid separation and identification to determine the quantity of tryptophan (Trp) in growing plant cells [75]. The Trp content was evaluated in the 5 d, 10 d cultured explants of Col-0 on E0, ET and EA. Based on the age of the culture, from 500 (5th day) to 100 (10th day) explants per repetition were collected to analyze Trp content. The experiment was carried out in triplicate. The fresh mass of explants was measured, and then the material was lyophilized by 24 h (Christ Alpha 1–4). After this process, probes were kept at -80 °C till the next phase of the procedure. Few extractions and developing solvents were tested. The method described below was chosen as the most efficient. At the beginning of the extraction procedure, probes were refrigerated and set on the ice. To extract free tryptophan from cells, 0.1 M HCl was used (1.5 µL/1 mg of fresh mass). The material was homogenized in extraction solvent, first by mechanical and later by ultrasonic homogenizer (VibraCell, Sonic Materials, 10 pulses 1s long, 10% amplitude). Probes were centrifuged after homogenization (15 min, 15,000 RPM at 4^o^C). 8 µL of supernatant was added to a TLC silica plate (DC-Fertigplatten ADAMANT 20 × 10 cm, Macherey-Nagel), on the same plate calibration curve for tryptophan (SIGMA) was prepared. After application, the TLC plate was left to dry for 1 h in the dark. The plate was developed in the horizontal chamber with the use of n-butanol: acetic acid: water (3:1:1) for 90 min (Baron, Economidis, 1963). The developed plate was left to dry (60 min). Next, the plate was sprayed with a solvent of 0.5% ninhydrin in methanol and incubated on a hotplate for 5 min at 90^o^C. A dry plate with spots was archived digitally for further analysis. TRP quantity in visualized spots was calculated with the use of CP Atlas 2.0 software [76]. The quantity of Trp was expressed as ug per mg of fresh tissue mass.

### Identification of insertional mutants and gene functional analysis

The seeds of insertional or chemically induced mutants were purchased as part of the T_3_ segregating lines. To identify homozygous mutants DNA was isolated from at least 24 individual T_3_ plants using a modified micro-CTAB method [77]. PCR reactions containing two gene specific primers and one insert-specific primer (http://signal.salk.edu/tdnaprimers.html) were then conducted (Additional file 1). The size of amplified products indicated the homo- or heterozygotic status of the plants, in terms of the insert presence within the gene of interest. The explant capacity for SE was evaluated in an IZE-derived culture that was induced for 21 days on an induction media and two parameters were calculated – SE efficiency, and productivity as described previously [59].

### Isolation of RNAs, reverse transcription, and RT-qPCR analyses

An RNAqueous^®^ Total RNA Isolation Kit (ThermoScientific) was used to isolate the total RNAs, respectively, from the 100 IZE explants induced on the different media for 10 days. The concentration and purity of the RNA were assessed using an ND-1000 spectrophotometer (NanoDrop). In order to control any DNA contamination, the RNAs were treated with RQ1 RNase-free DNase I (Promega) according to the manufacturer’s instructions. The first-strand cDNA was produced in a 20 µL reaction volume using a RevertAid First Strand cDNA Synthesis Kit (Fermentas). The reverse transcription product was diluted with water at a 1:4 ratio and 2.5 µL of this solution was used for the RT-qPCR reactions (Additional file 1). The relative RNA levels were calculated and normalized to the internal control of the *AT4G27090* (*TIN*) gene-encoded 60 S ribosomal protein [78]. The relative expression level was calculated using 2^−*ΔΔC*T^ where ∆∆*C*_T_ represented ∆*C*_T_^reference condition^ − ∆*C*_T_^compared condition^. The plant tissues for the gene expression analysis were produced in three biological replicates, and two technical replicates were analyzed [50].

### Data availability

The RNA-seq data presented in this publication have been deposited in NCBI’s Gene Expression Omnibus (GEO) and are accessible through GEO Series accession number GSE255229. All other data is available upon reasonable request.

## Results

### Experimental design

A standard method for SE induction in Arabidopsis involves the treatment of the explants, immature zygotic embryos (IZEs), with a synthetic auxin, 2,4-D [59]. We showed that, similar to auxin, explant treatment with an inhibitor of histone deacetylases TSA, results in SE induction [50], suggesting the role of Hac in the embryogenic reprogramming of plant somatic cells. Here, the RNA-seq-generated transcriptomes of the TSA- and auxin-induced cultures were analyzed to get insights into the Hac-related mechanism of SE induction.

In brief, the IZE explants at the cotyledonary stage of development were cultured on two alternative SE-induction media, ET and EA, supplemented with TSA (1 µM) and auxin (5 µM 2,4-D), respectively. In both SE cultures, the first somatic embryos became visible on the adaxial side of the IZE cotyledons on the 8-10th day. In contrast, the explants that were cultured on a non-embryogenic medium (E0) developed into seedlings (Additional file2). The EA- and ET-cultured explant tissues were sampled for RNA-seq analysis at two culture points representing the early (5th day) and the advanced (10th day) stage of SE induction. The control combinations involved the explants cultured on E0 for 5, and 10 days. In total, six experimental combinations in three replicates were used in RNA-seq analysis (Additional file3).

### General characteristics of ET and EA transcriptomes

#### PCA analysis

**Fig. 1:**
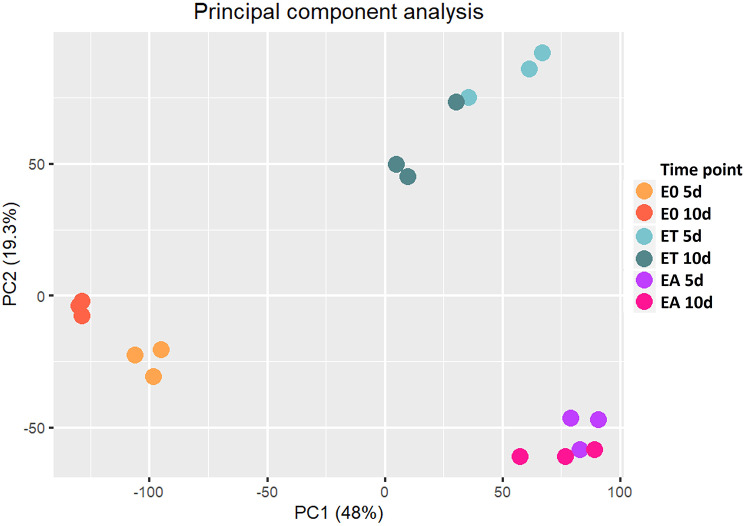
Principal Component Analysis (PCA) of eighteen RNA-seq libraries. The analysis demonstrates a clear separation of gene expression profiles in embryogenic, TSA (ET) and auxin (EA) induced, and non-embryogenic (E0) cultures at 5 d and 10 d. Expression data from three independent biological replicates were analyzed. Biological replicates are depicted as dots of the same color

The Principal Component Analysis (PCA) and Hierarchical Cluster Analysis demonstrated a high concordance between replicates of the same experimental combination (medium type x culture time point) (Fig. 1; Additional file4). The PCA revealed that 48.0% and 19.3% of the transcriptome variation accounted for principal components 1 (PC1) and 2 (PC2), respectively. PC1 separated the samples treated with TSA (ET) and auxin (EA) from those induced on the control E0 medium. PC2 stratified the transcriptomes according to TSA treatment.

The PCA results implied that transcriptomes representing the embryogenic (ET and EA) vs. non-embryogenic (E0) cultures were distinctly different at both of the analyzed culture time points (5 and 10 d). Moreover, the embryogenic cultures induced on ET and EA medium tended to overlap, indicating similarities between TSA and the auxin (2,4-D)-induced transcriptomes. These results suggest that the regulatory pathways controlling SE induction in response to TSA and auxin treatment might share some genetic factors.

### Gene expression profiling in SE-induced vs. control explants (DEGs-ET/E0 and DEGs-EA/E0)

**Fig. 2:**
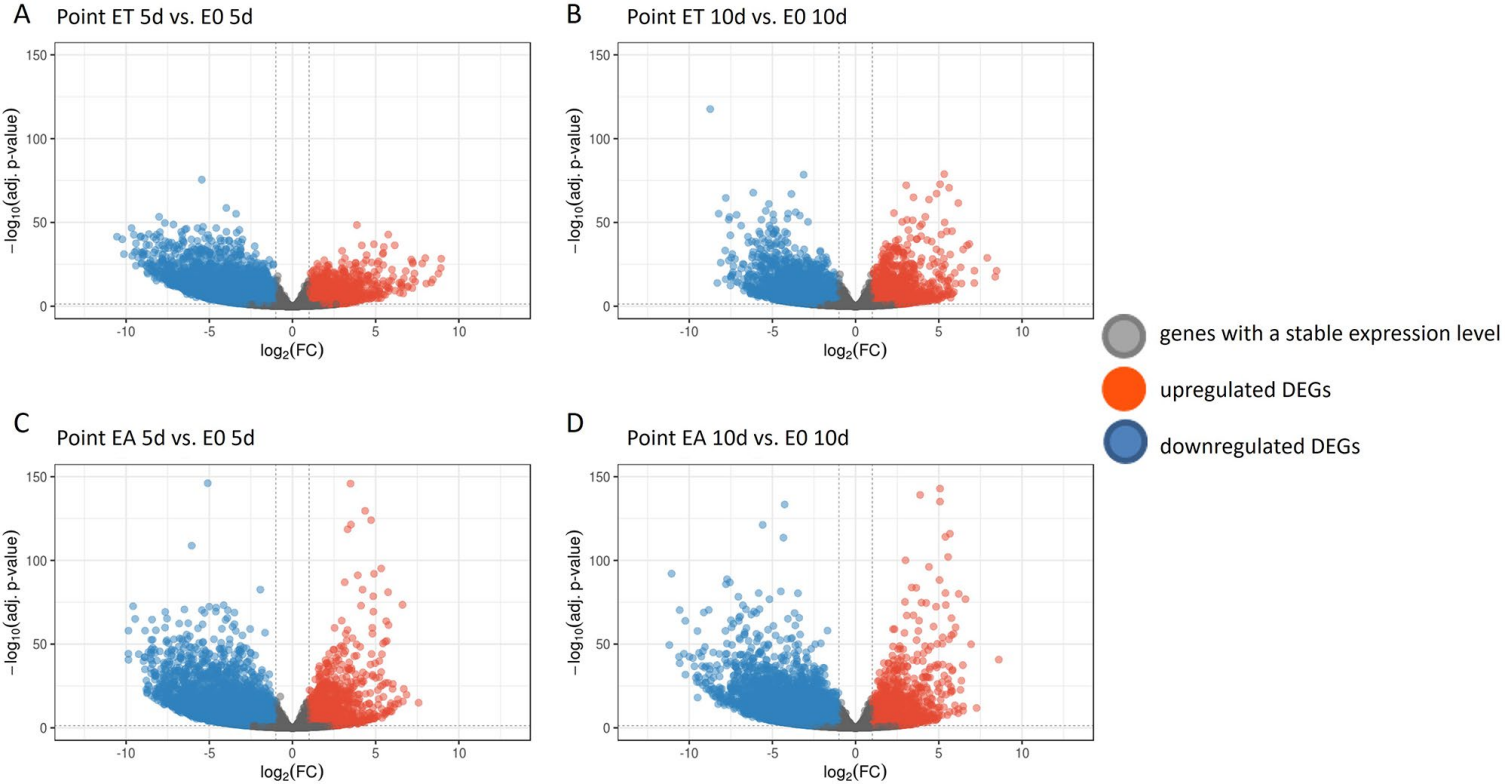
Volcano plot of differentially expressed genes (DEGs) in ET (**A**, **B**) and EA (**C**, **D**) embryogenic cultures on the 5th (**A**, **C**) and 10th (**B**, **D**) day. ET and EA - embryogenic cultures induced with TSA and auxin, respectively. The horizontal line at false discovery rate (FDR) = 0.05; the vertical line at |log2FC| = 1

**Fig. 3:**
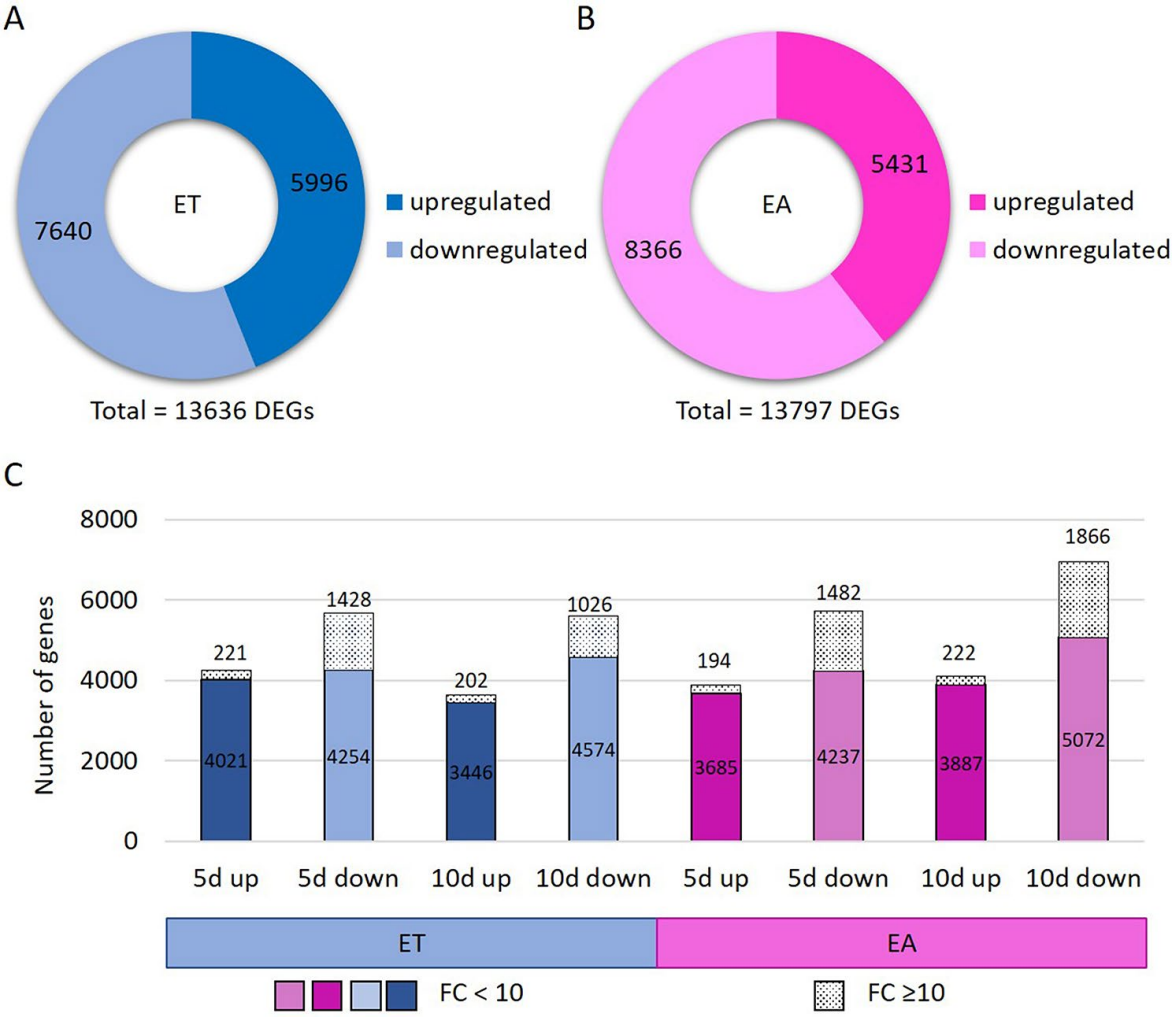
The number of up- and downregulated DEGs in embryogenic cultures induced in explants on an ET medium with TSA (**A**), and an EA medium with auxin (**B**). The number of moderately (FC < 10) and highly deregulated (FC ≥ 10) genes (**C**)

Next, we compared transcript levels in embryogenic (ET and EA) vs. non-embryogenic (E0) cultures to identify differentially expressed genes (DEGs) responsive to TSA (DEGs-ET/E0) and auxin (DEGs-EA/E0) treatment. DEGs were detected using DESeq2 [70]. Raw P values were adjusted for multiple comparisons according to Benjamini and Hochberg’s method controlling the False Discovery Rate (FDR). Significantly regulated genes were selected under a threshold of FDR ≤ 0.05 and |log2FC| ≥ 1. Volcano plots were used to illustrate the distribution of the log2FC and FDR within the analysed gene sets (Fig. 2). Using these criteria lists of DEGs-ET/E0 and DEG-EA/E0 genes of differential expression in ET (Fig. 3A) and EA (Fig. 3B) cultures, respectively, were generated in comparison to E0 culture (Additional file 5). The results indicated a similar fraction of DEGs in both SE cultures; 44.4% (13 636) and 44.9% (13 797) in ET and EA, respectively. Moreover, downregulated transcripts were found more frequently than upregulated in both SE induction treatments. Accordingly, 56.0% and 60.6% of DEGs showed a significantly decreased expression in response to TSA (DEGs-ET/E0) and auxin (DEGs-EA/E0) treatment, respectively. Noteworthy, at least one-fifth of the downregulated DEGs in embryogenic cultures showed a high decrease in transcript abundance by at least tenfold (FC < 10) in both ET and EA treatments. More specifically, this criterion fulfilled ~ 26% (1428) and ~ 27% (1866) of downregulated DEGs in ET and EA cultures, respectively (Fig. 3C). Substantially less frequent were gene transcripts of highly increased (FC ≥ 10) expression, which accounted for ~ 5% of the upregulated DEGs; 221 and 222 in ET and EA culture, respectively.

**Fig. 4:**
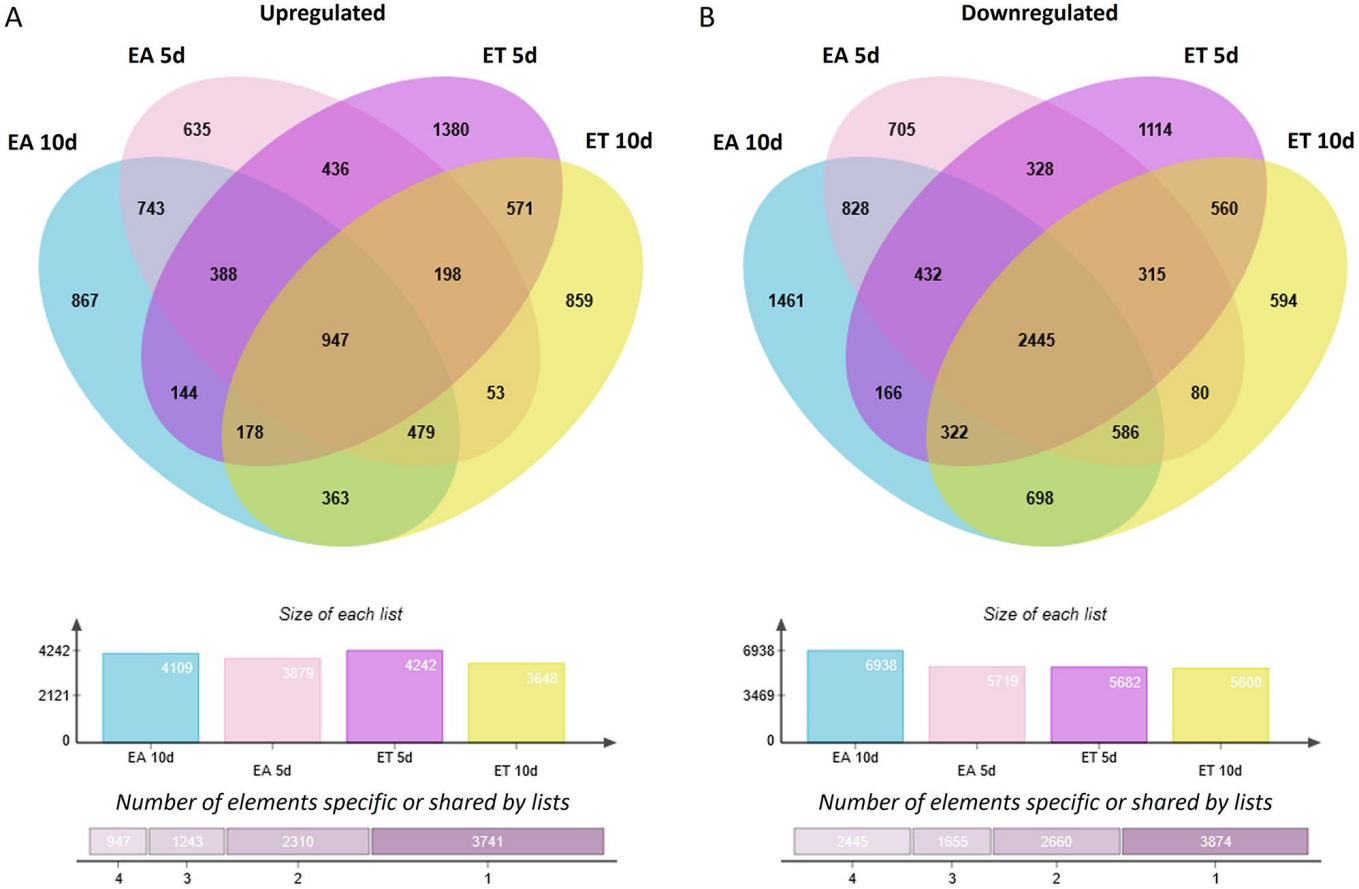
An overlap between up- (**A**) and down- (**B**) regulated DEGs identified in ET (ET vs. E0) and EA (EA vs. E0) embryogenic cultures of explants. ET – SE induced with TSA; EA – SE induced with auxin; E0 – non-embryogenic control culture

To get more insights into the differences between the TSA- and auxin-induced transcriptomes, we juxtaposed DEGs identified in ET (DEGs-ET/E0) and EA (DEGs-EA/E0) cultures (Fig. 4). The analysis of the up- (Fig. 4A) and down- (Fig. 4B) regulated DEGs revealed that the majority of them were deregulated in response to one of the SE treatments, ET or EA. Accordingly, 37.2% (5078) and 38.0% (5239) DEGs were exclusively deregulated in response to TSA or 2,4-D treatment. In this group, DEGs upregulated only in response to TSA were more frequent; 20.6% (2810) and 16.5% (2245) genes responded specifically to TSA and auxin, respectively (Fig. 4A). In contrast, genes of treatment-specific downregulation were more numerous in EA culture, 21.7% (2994) and 16.6% (2268) transcripts showed decreased expression exclusively in EA or ET culture, respectively (Fig. 4B).

Gene Ontology (GO) term analysis (Additional file6) revealed that a significant fraction (29%; 827/2810) of the ET-specifically upregulated transcripts were involved in response to different stimuli such as light (131), temperature (107), biotic (220), abiotic stimulus (349), and other stress response (536). In contrast, the DEGs of auxin-specific upregulation were predominantly related to the nucleobase-containing compound metabolic process (21.6%; 485/2245), i.e., transcription, translation, splicing, metabolism of RNA, and chromatin reorganization. The TSA-specific downregulated genes were mainly attributed to the transport of ions, amides, proteins, nitrogen, and the metabolism of proteins and lipids. The function of auxin-specific downregulated genes was associated with response to stimuli and stress (939), and photosynthesis (106). Altogether the results indicated numerous genes of diverse biological functions, including those involved in cellular housekeeping processes and responses to stress and various external stimuli, to be deregulated explicitly in response to TSA or auxin treatment. The strong deregulation of stress-related genes in response to TSA implies the involvement of histone deacetylation in regulating stress responses in plants.

Next, we aimed to identify genes of a similar expression profile in TSA- and auxin-induced SE. To this end, we focused on DEGs that were up- or downregulated during the early (5 d) and advanced (10 d) stages of SE in both ET and EA cultures. The analysis revealed 1746 up- and 3471 downregulated DEGs at the early and late SE, respectively (Fig. 4A, B). These DEGs involved 947 up- and 2445 downregulated in ET and EA culture during both SE stages (5 and 10 d). Altogether, the results indicated over 2.5 times more transcripts of constantly decreased vs. increased expression in embryogenic culture, suggesting that extensive negative regulation of genes is associated with the embryogenic transition of a somatic cell transcriptome.

GO term analysis of DEGs that were upregulated in both stages of ET and EA cultures indicated numerous transcripts involved in the cell cycle (171), DNA replication (55) and repair (93), chromosome organization (113), and RNA virus-induced gene silencing (27). These transcripts contribute to basic molecular processes, including cell divisions, growth, and differentiation of key roles in cell reprogramming (Additional file7). The SE-upregulated DEGs were also annotated to other indicated roles in embryogenic transition processes, including auxin response (39), floral organ development, and embryogenesis (139 genes). Within the upregulated in SE DEGs, we identified *TF* genes of an essential function in embryogenic development, such as *LEC1*, *LEC2*, *AUXIN RESPONSE FACTORS* (*ARFs*), *INDOLE-3-ACETIC ACID INDUCIBLE* (*AUX/IAA*), *PHABULOSA* (*PHB*), *PHAVOLUTA* (*PHV*), *SOMATIC EMBRYOGENESIS RECEPTOR-LIKE KINASE 1* (*SERK1*), *PIN-FORMED* (*PINs*), *WOXs*, *EMBRYO DEFECTIVE* (*EMBs*). DEGs of downregulated expression during SE in ET and EA cultures were annotated with cell wall organization (145) and differentiation of organs, tissues, and cells (e.g., root 96; epiderm 52, root hair 30, trichoblast 40). Numerous genes involved in the biosynthesis and metabolism of JA (16) and xyloglucan and xylan metabolism (41) were also identified (Additional file7).

In summary, the GO term analysis showed that the SE-upregulated DEGs were directly linked with plant reproduction and embryogenic processes, while the SE-downregulated DEGs mostly represented differentiation and cell specification processes. Similarly deregulated in ET and EA cultures, DEGs might reveal a set of common and essential, regardless of inducer, SE regulators that involve *TFs*, including those of documented already function in SE (such as *LECs*, *SERKs*, *WOXs*), and candidate genes related to plant reproduction and zygotic embryogenesis.

### *TF* genes of SE-deregulated expression

**Fig. 5:**
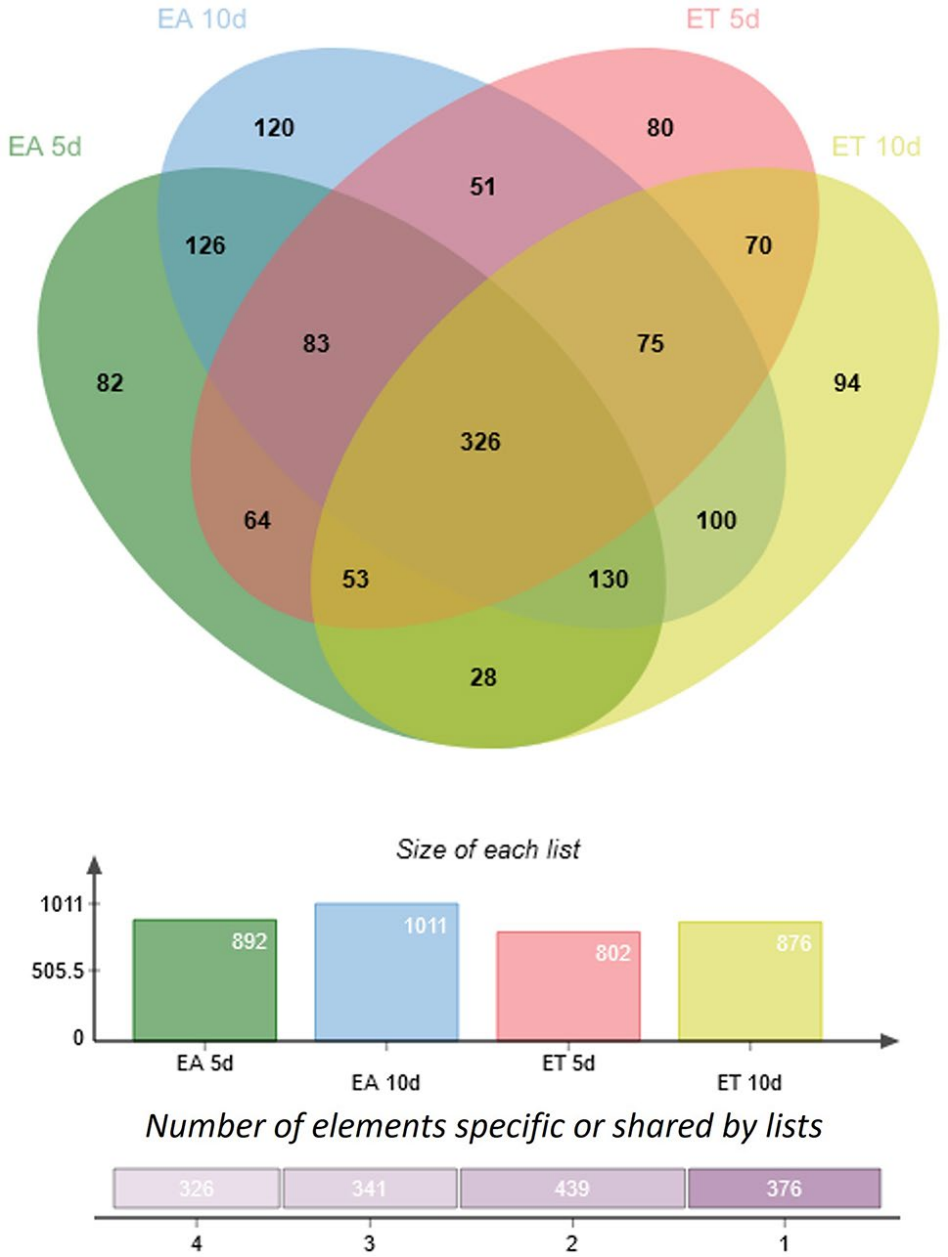
Differentially deregulated *TF* genes in SE induced on TSA (ET) and auxin (EA) media. Venn diagram showing *TF*-DEGs identified by comparison of genes expressed in ET and EA embryogenic cultures to transcripts in E0, non-embryogenic culture

Given the central role of regulatory genes in the genetic network controlling SE induction, we searched for *TFs* within DEGs of SE-modulated expression. The analysis indicated that more than half of the analyzed 2178 *TF* genes were deregulated in response to both SE treatments. Accordingly, 1154 (53%) and 1238 (57%) TF transcripts were deregulated in response to TSA- and auxin-treatment, respectively (Fig. 5).

In general, most of the TF-DEGs showed similar trends in expression profiles in ET and EA culture (Additional file8). We also searched for *TF* gene families with TSA- and auxin-specific deregulation, and we identified eleven *TF* families whose members were up- (*C2C2-YABBY*, *CSD*, *DBP*, *HRT*, *Sigma70-like*, and *VOZ*) and down- (*BBR/BPC*, *HMG*, *Jumonji*, *MED7*, *SNF2*) regulated exclusively in response to TSA. Moreover, ten *TF* gene families of an auxin-specific pattern of deregulation, including *BSD*, *EIl*, and *MBF1* (upregulation) and *ARF*, *C2C2-YABBY*, *CSD*, *FAR1*, *IWS1*, *SWI/SNF-BAF60b*, *ULT* (downregulation) were identified (Fig. 5; Additional files 8 and 9). Importantly, we found most of the central SE-regulators, including *LEC1*, *LEC2*, *FUS3*, *ABI3*, *PHB*, *PHV*, *AGL15*, and most of the *WOX* and *PLT* gene family members upregulated in response to both treatments, TSA and auxin (Additional file10). A particularly high (FC 6 to 51) increase of the *LAFL* and *WUS/WOX* family members’ expression in response to TSA, including *LEC1*, *LEC2*, *FUS3*, *WUS*, *WOX3*, and *WOX8* suggests the role of Hac in their regulation.

### Genes differentially expressed in ET vs. EA culture (DEGs-ET/EA)

**Fig. 6:**
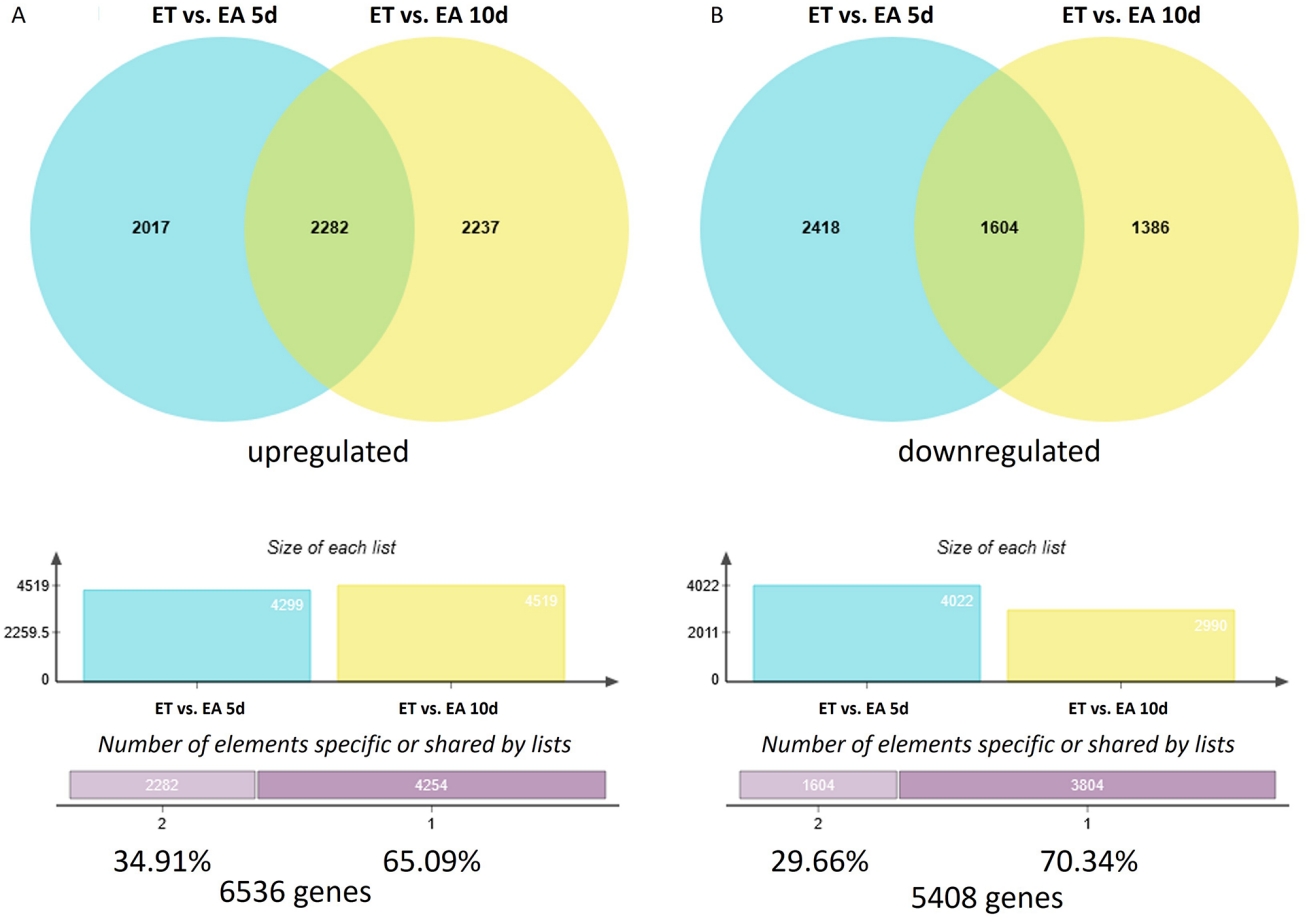
DEGs of up- (**A**) and down-regulated (**B**) expression in ET compared to EA culture (DEGs-ET/EA) on the 5th and 10th day of SE induction

Besides distinguishing SE-DEGs relevantly to non-embryogenic (E0) culture (DEGs-ET/E0 and DEGs-EA/E0), we also identified transcripts of different expression levels in ET vs. EA cultures, DEGs-ET/EA (Additional file11). To this end, the up- (Fig. 6A) and down- (Fig. 6B) regulated ET/E0 and EA/E0 DEGs were juxtaposed, and the DEGs-ET/EA of up-, and downregulated expression in the early (5 d) and advanced (10 d) stage of SE were indicated. The results evidenced that 59% (8058/11,944) of the DEGs-ET/EA showed different expression levels in ET vs. EA at one of the culture points (5 or 10 d), and one-third (3886/11,944) had different transcript levels at both SE stages (5 and 10 d). Noteworthy, the majority 54.7% (6536/11,944) of the DEGs-ET/EA, displayed a higher transcription level in ET vs. EA culture.

GO term analysis of the upregulated DEGs-ET/EA revealed numerous transcripts related to stimuli (1857/6536) and stress responses (1170/6536) (Additional file12). Insights into the biological function of the downregulated DEGs-ET/EA indicated frequent transcripts related to biological regulation (1250), development (674), and regulation of hormone levels (90) (Additional file12). A set of genes differentially expressed in ET vs. EA provides candidate genes of the Hac-controlled expression in SE.

### Insights into functional categories of SE-DEGs

#### Auxin-related DEGs

In line with the fundamental role of auxin responses in the SE induction mechanism, genes involved in various aspects of auxin action, including biosynthesis, signaling, and polar transport of auxin, were found frequent within SE-DEGs induced with TSA and auxin.

### DEGs related to auxin biosynthesis

#### Tryptophan (Trp)-dependent IPA-pathway of indole-3-acetic acid (IAA) biosynthesis

The genes encoding aminotransferases (*TAA1* and *TARs*) and monooxygenases (*YUCCA*) of critical role in the IPA (indole-3-pyruvic acid) auxin biosynthesis pathway were substantially deregulated in SE (Additional file13–1 A). Within aminotransferase genes, transcription of the *TAA1* gene was increased significantly up to over five (FC 5.6) and two (FC 2.1) fold in the ET and EA culture, respectively. Analysis of eleven *YUC* genes (*YUC1-11*) indicated that different YUC monooxygenases might control IAA production in TSA- and auxin-induced SE. Two *YUC* genes, *YUC4* and *YUC10* were upregulated in TSA-induced SE, of which *YUC10* transcripts showed the highest accumulation, up to 7.5 and 46.6 FC in the early (5 d) and advanced (10 d), respectively, stage of the ET culture. Auxin treatment resulted in the upregulation of four other *YUC* transcripts, *YUC3*, *7*, *8*, and *9*, of which *YUC3* (FC up to 29) and *YUC7* (FC up to 35) showed a particularly high level of transcript accumulation in both culture stages.

#### Tryptophan biosynthesis

Since tryptophan (Trp) is a substrate for IPA production, transcripts of numerous (20) genes engaged in the biosynthesis of this amino acid were analyzed in ET and EA embryogenic cultures (Additional file13–2 A). The results revealed that different Trp-biosynthesis genes showed a rapid and transient increase of expression in the early stage (5 d) of SE induced on ET and EA medium. TSA treatment resulted in the upregulation of twelve Trp-biosynthesis genes (*ANTHRANILATE SYNTHASE ALPHA SUBUNIT 1*, *2*; *ASA1*,*2*, *ASA PUTATIVES*, *ANTHRANILATE SYNTHASE BETA SUBUNIT 1*, *2*; *ASB1*,*2*, *INDOLE-3-GLYCEROL PHOSPHATE SYNTHASE*; *IGPS*, *IGPS PUTATIVE*, *PHOSPHORIBOSYLANTHRANILATE ISOMERASE 1*, *3*; *PAI1*,*3*, *PHOSPHORIBOSYLANTHRANILATE TRANSFERASE 1*; *PAT1*, *TRYPTOPHAN SYNTHASE ALPHA CHAIN 1*; *TSA1*) while three of them (*ASA1*, *ASA PUTATIVE*, *PAI3*) showed also increased expression in response to auxin treatment (Additional file 13–2 A). The majority (9/12) of the Trp biosynthesis-related genes showed distinctly higher (1.62–2.0) accumulation of transcripts in the explants cultured on ET vs. EA medium (Additional file13-2 B). The results pointed to the role of the Hac regulation in SE-related Trp biosynthesis.

#### IAA and Trp content

**Fig. 7:**
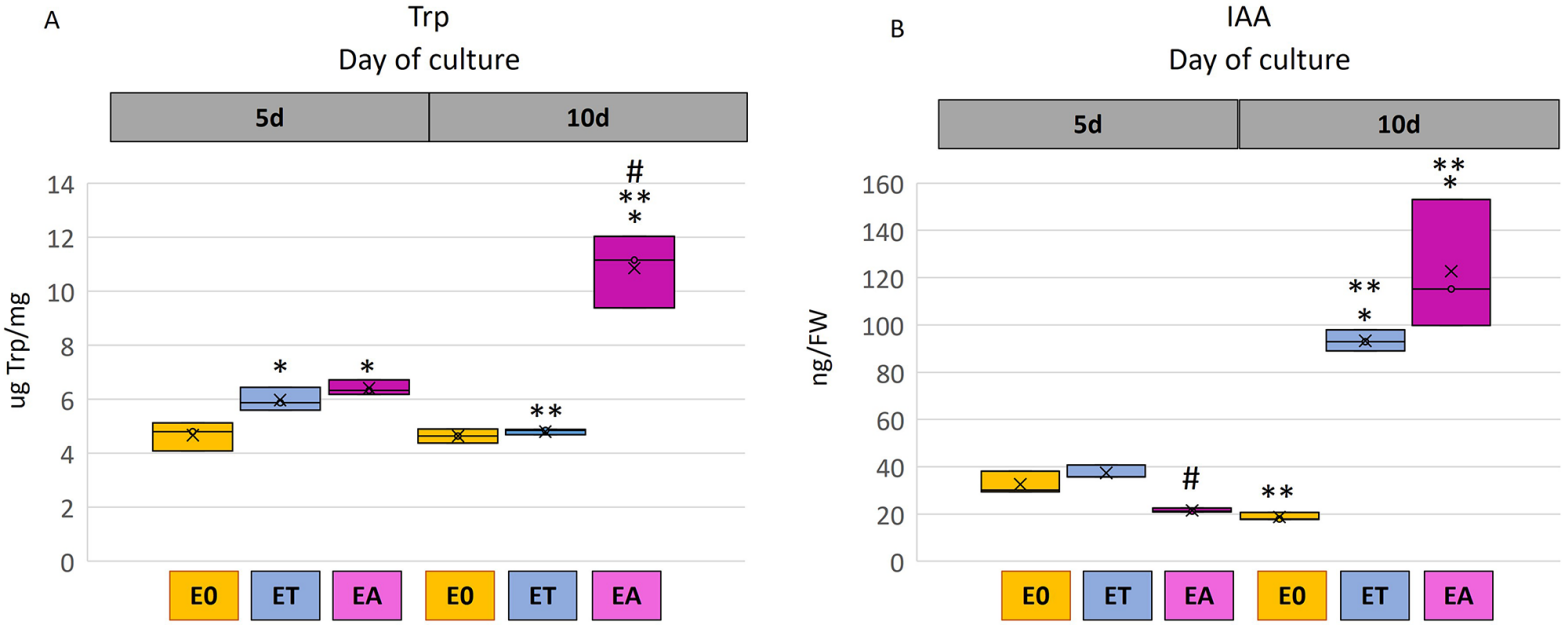
Level of Trp (**A**) and IAA (**B**) in TSA- (ET) and auxin- (EA) induced somatic embryogenesis and seedling development on control E0 medium. A two-way ANOVA analysis (*p* < 0.05) followed by Tukey’s HSD (*p* < 0.05) was used to determine any values that were significantly different. Significant differences between embryogenic (ET and EA) and control (E0) culture on the same day (*); the 5th and 10th day of the culture (**); ET and EA on the same day of culture (#); (*n* = 3; means ± SD are given). FW – fresh weight

In line with the upregulation of Trp- and IAA-biosynthesis genes, we indicated the elevated abundance of tryptophan (Trp) and IAA in the embryogenic (ET and EA) vs. control (E0) cultures (Fig. 7). More specifically, a 1.5-fold increase of Trp content was indicated in the early SE stage of TSA and auxin-induced explants (Fig. 7A). In the advanced SE (10 d), a higher Trp content was found exclusively in auxin-treated culture. The analyses also revealed that a level of IAA was significantly increased (up to 5- 6-fold) at the advanced stage of SE induction (10 d) in both ET and EA cultures (Fig. 7B).

#### *NITRILASE* (*NIT*) genes involvement in SE

**Fig. 8:**
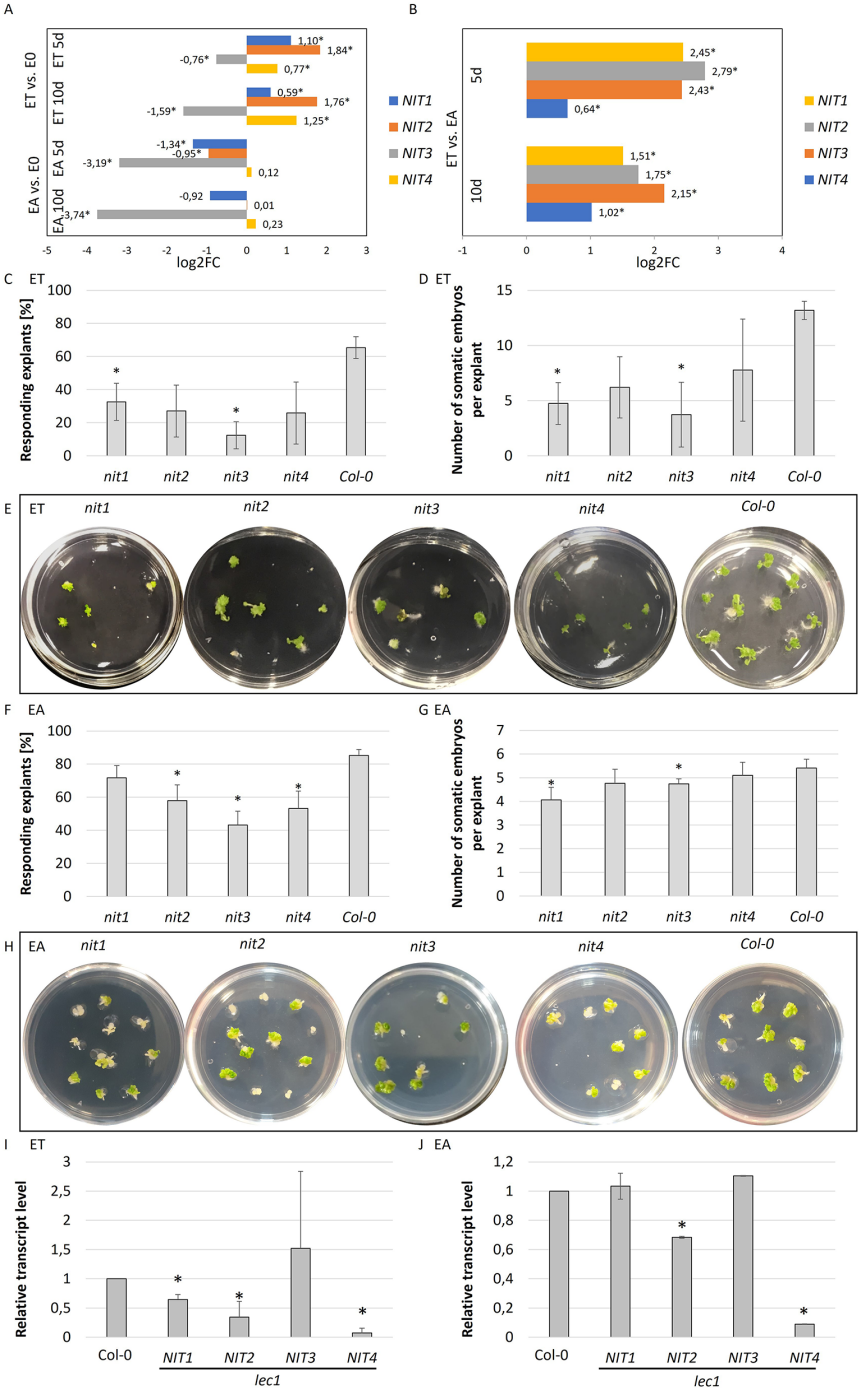
The involvement of *NITRILASE* (*NITs*) genes in SE induction. Differential expression level of *NIT1-4* genes in explants cultured on embryogenic (ET, and EA) and control E0 medium for 5 and 10 days. Values represent the relative expression level (log2FC) in the ET vs. E0, EA vs. E0 (**A**), and ET vs. EA (**B**). Data from the RNA-seq analysis are given. Wald’s exact test was used to identify any differentially expressed genes (DEGs) under a p-value adjustment (*p* < 0.05) for multiple comparisons with the Benjamini-Hochberg False Discovery Rate (FDR) correction. (*) significant differences between the gene expression in E0 vs. ET or EA at the same age, or between ET and EA at the same age. The impaired embryogenic response of *nit* (*nit1-4*) mutants (**C** –**H**). Analysis of the effectivity (**C**, **F**) and productivity (**D**, **G**) of SE in the culture of *nit* and WT (Col-0) explants cultured on ET (**C**, **D**, **E**) and EA medium (**F**, **G**, **H**). (*) significant differences between *nit* mutants and Col-0 (**C**, **D**, **F**, and **G**) (*p* < 0.05, Student’s t test). Expression of *NIT1-4* genes in *lec1* explants culture induced on ET (**I**) and EA (**J**) medium for 10 days. (*) significant differences between the gene expression in *lec1* and WT (Col-0); (A two-way ANOVA analysis (*p* < 0.05) followed by Tukey’s HSD (*p* < 0.05) was used to determine any values that were significantly different

Nitrilases (NITs) were postulated to catalyze auxin biosynthesis via an alternative to the IPA-route pathway [79, 80]. The results indicated three (*NIT1*, *2*, and *4*) out of four *NIT* genes encoded in Arabidopsis were upregulated (1.5 to 3.6 fold) in the TSA-induced explants (ET) in comparison to the control (E0) culture (Fig. 8A, B). In contrast to ET, *NIT1* and *2* were downregulated in the auxin-induced SE (EA vs. E0). The expression level of *NITs* was significantly higher (1.6 to 5.5 times) in the TSA- vs. 2,4-D-induced SE (Fig. 8B).

To further explore the involvement of *NIT* genes in embryogenic induction, the embryogenic potential of the *nit* (*nit1-4*) mutants was analyzed in ET (Fig. 8C, D, E) and EA (Fig. 8F, G, H) cultures. We indicated that all *nit1-4* mutants were impaired in their embryogenic response in EA culture, and two of them, *nit1* and *nit3* in ET culture. We also analyzed the expression of *NIT*s in *lec1* explants during SE induction, taking into account the key regulatory role of LEC1 TF in auxin biosynthesis in SE induction [81, 82]. The substantial downregulation of *NIT1*, *2*, and *4* transcripts in the *lec1* mutant culture suggests that LEC1 might positively regulate *NITs’* expression in SE-induced explants (Fig. 8I, J).

### DEGs involved in auxin signaling

Most of the genes encoding the main components of the auxin signaling pathway, including *AUX/IAA* and *ARFs*, were extensively deregulated in the SE-induced explants. Accordingly, out of 29 *AUX/IAA* genes, 8 and 19 were significantly upregulated in ET and EA culture, respectively (Additional file13–3 A). The upregulated (1.6 to 43.4 fold) in both ET and EA culture *AUX/IAA* genes involved *IAA 1*, *20*, *29*, *30*, and *33.* In general, the *AUX/IAA* showed a significantly higher expression level in response to auxin than TSA (Additional file13-3 B).

Like *AUX/IAA*, most of the 23 *ARF* genes of Arabidopsis showed extensive deregulation in the SE-induced explants (Additional file13–4 A). Nine *ARF*s, including *ARF3*, *4*, *5*, *6*, *8*, *10*, *17*, *18*, and *19*, were significantly upregulated (1.2 to 15.2 fold) in response to both SE treatments. In particular, *ARF5* was highly upregulated (up to 15.2-fold) in auxin treatment. Most of the *ARF*s responded similarly to ET and EA media and were up- or downregulation in both treatments. In contrast, *ARF11* showed opposed changes in expression in different treatments and the gene was upregulated (3.0 fold) and downregulated (8.2 fold) in ET and EA cultures, respectively. Like *Aux/IAA*, most SE-deregulated *ARF*s showed higher expression in EA than ET culture except for *ARF 4*, *11*, and *18*, whose transcription level was higher in TSA-treated explants (Additional file13-4 B).

### DEGs related to polar auxin transport

Insight into the DEGs related to the auxin transport pathway showed differential expression of numerous genes encoding the influx and efflux carriers of IAA, including *PINs*, *AUXIN1/LIKE-AUX1* (*AUX*/*LAX*) in both TSA- and 2,4-D-induced SE (Additional file13–5 A). However, individual *PIN* and *LAX* genes indicated differences in gene expression patterns on ET and EA media. Accordingly, out of twelve genes analyzed in this group, eight and five genes were upregulated in ET and EA medium, respectively (Additional file13–5 A). In both SE treatments, the increased expression (1.4 to 21 fold) showed *PIN1*, *3*, *6*, and *LAX1*, *2*. In contrast, *PIN2*,*5* and *LAX3* genes were downregulated (3 to 135 fold) in ET and EA cultures. In general, transcripts of the auxin transport genes accumulated at a lower level (2 to 11.6 fold) in ET vs. EA culture (Additional file13-5 B).

### Stress-related DEGs

**Fig. 9:**
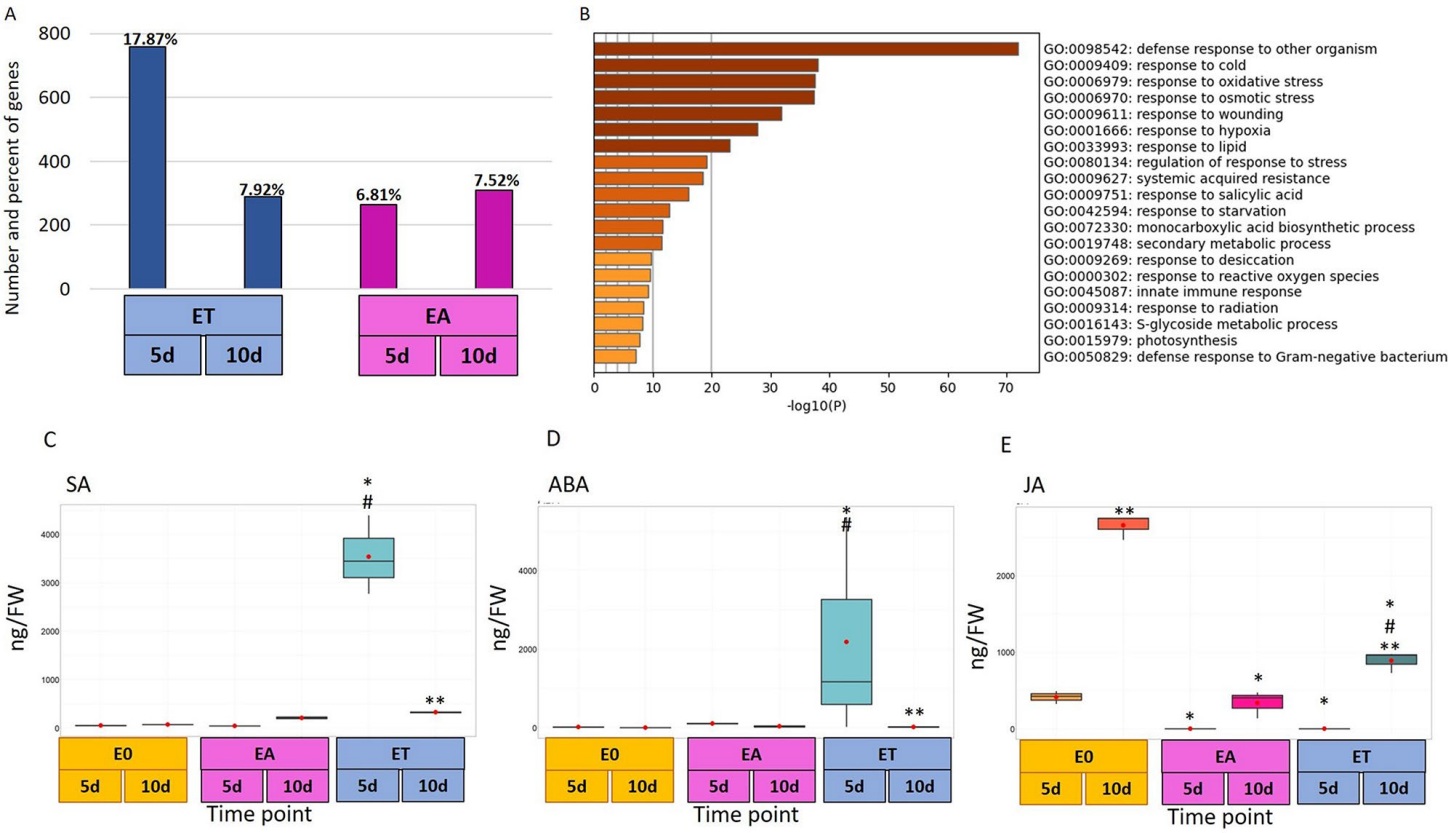
The involvement of stress in somatic embryogenesis induction. The number and percent of stress-related upregulated DEGs in TSA (ET) - and auxin (EA) - induced embryogenic cultures on 5th and 10th day (**A**). GO analysis of stress-related transcripts with higher expression levels in ET than EA culture (ET vs. EA) (**B**). A level of stress-related phytohormones, SA (**C**), ABA (**D**), JA (**E**) in somatic embryogenesis induced on ET or EA medium, and seedling development on E0 medium. A two-way ANOVA analysis (*p* < 0.05) followed by Tukey’s HSD (*p* < 0.05) was used to determine any values that were significantly different. Significant differences between: embryogenic (ET and EA) and control (E0) culture on the same age (*); the 5th and 10th day of the culture (**); ET and EA on the same day of culture (#) (*n* = 3; means ± SD are given). FW – fresh weight

The commonly accepted role of stress factors in the mechanism controlling SE induction (reviewed in [83]) motivated us to get insights into DEGs related to stress responses. Closer inspection of the upregulated DEGs indicated a high number (758) of stress-related transcripts that were massively accumulated at the early stage (5 d) of the TSA-induced SE (Fig. 9A). The stress-related transcriptome was also activated in the EA culture, although less intense than in ET. The number (264) of the stress-related DEGs that were upregulated in the early response to auxin treatment (5 d EA) was almost three times lower than in the relevant stage of TSA-induced culture (5 d ET). These results demonstrated that TSA treatment might specifically activate the stress-related transcripts. In line with this assumption, GO analysis of transcripts with higher expression levels in ET than EA culture (ET vs. EA) revealed numerous genes (462) involved in stress response (Fig. 9B). In this group, the genes involved in systemic acquired resistance and responses to biotic and abiotic stresses, including cold, hypoxia, osmotic, and oxidative stress were the most highly represented.

Insights into stress-related DEGs in SE cultures revealed transcripts encoded the critical enzymes of the stress hormone metabolism, including salicylic (SA), abscisic (ABA), and jasmonic (JA) acid. In line with RNA-seq results, we indicated significant differences in SA, ABA, and JA levels between the embryogenic and non-embryogenic (ET vs. E0, EA vs. E0) and within the embryogenic (ET vs. EA) cultures (Fig. 9C-E). In particular, a high accumulation of two stress hormones SA and ABA, up to 68- and 179-fold relevantly, was found in the explants induced with TSA for five days (Fig. 9C, D). In contrast, JA showed distinctly decreased accumulation in the embryogenic (ET and EA) compared to the control E0 culture (Fig. 9E).

Within the DEGs that might contribute to a high accumulation of SA in TSA-induced explants, we found the *ISOCHORISMATE SYNTHASE 1* (*ICS1*) gene engaged at the critical step of the SA biosynthetic pathway substantially upregulated (11.5 fold) in the early stage (5 d) of the TSA-induced SE (Additional file14–1 A, B). In addition to *de novo* biosynthesis, the increased SA content might also result from the reduced conversion of SA to SA derivatives. In support of such possibility, the genes involved in the production of methyl salicylate (MeSA) derivate from SA, including *METHYL ESTERASEs* (*MES1*, *2*,* 7*, *9*) were significantly downregulated (5.1 to 40.5 fold) in ET culture.

According to the TSA-increased ABA accumulation, transcription of *NINE-CIS-EPOXYCAROTENOID DIOXYGENASE 6* (*NCDE6*) involved in ABA biosynthetic pathways was elevated (64 fold) in ET vs. E0 culture (Additional file14–2 A). In addition, the *BETA GLUCOSIDASE 1* (*BG1*) gene encoding the esterase that cleaves the ABA glucoside to active ABA was distinctly upregulated (6.2 fold) in ET culture. In addition, the downregulation of the *CYP707A4* gene engaged in ABA catabolism might also contribute to the accumulation of ABA in ET culture. Thus, transcriptomic data suggest that the accumulation of ABA in the TSA-treated explants might result from an increase of *de novo* hormone biosynthesis, the release of active form from storage conjugates, and a decrease in ABA catabolism.

Analysis of DEGs revealed also genes involved in the biosynthesis and catabolism of JA in SE (Additional file14–3 A). Most of the genes related to JA biosynthesis were downregulated in the embryogenic cultures, which may explain the reduction in JA content in the SE process. Accordingly, the genes of significantly decreased expression in SE culture involved *LIPOXYGENASE 2* (*LOX2*), *ALLENE OXIDE SYNTHASE* (*AOS*), *ALLENE OXIDE CYCLASEs* (*AOC1*,* 2*,* 3*), *OXOPHYTODIENOATE-REDUCTASE 3* (*OPR3*), *SULFOTRANSFERASE 2 A* (*AtST2a*), and *ACYL-COA OXIDASE 2* (*ACX2*). Congruently to a higher JA level in ET than EA culture, the transcription level of genes involved in JA biosynthesis (*LOX2*, *AOS*, *AOC1*,*3*, *OPC-8:0 CoA LIGASE 1*; *OPLC1*) vs. JA metabolism (*JASMONIC ACID CARBOXYL METHYLTRANSFERASE*; *JMT*, *AtST2a*) was higher and reduced, relevantly, in ET vs. EA (Additional file14-3 B).

### DEGs involved in the specification of the organ polarity

We found that explant orientation on the medium affected the effectivity of somatic embryo production and the explant cotyledons placed adaxial side up on SE induction media (ET and EA) showed a much higher (up to 5 to 2 fold) embryogenic response compared to the explants oriented opposingly, i.e., the adaxial side down ([50]; Additional file15). To get insights into the genetic mechanism of adaxial-abaxial asymmetric SE response, we searched for transcripts related to the polarity of cotyledon and leaf within SE-DEGs. We identified seven TFs-encoding genes related to organ polarity of significantly upregulated (1.5 to 51.3 FC) expression in ET culture (Additional file16-1-3). Transcripts of four genes, including *RETARDED GROWTH OF EMBRYO 1* (*RGE1*), *ULTRAPETALA 2* (*ULT2*), *HOMEOBOX PROTEIN 32* (*HB32*), and *C-REPEAT BINDING FACTOR 1* (*CBF1*), showed higher, from 2.3 for *HB32* to 107-fold for *RGE1*, expression in ET vs. EA culture. Two of them, *HB32* and *ULT2*, showed opposed expression profiles in the TSA- vs. 2,4-D-induced explants, the up- vs. downregulation, relevantly. Moreover, auxin treatment inhibited *KANADIs* (*KAN1*,*3*,*4*) and *YABBY3* (*YAB3*) expression (Additional file16–4 A, B).

**Fig. 10:**
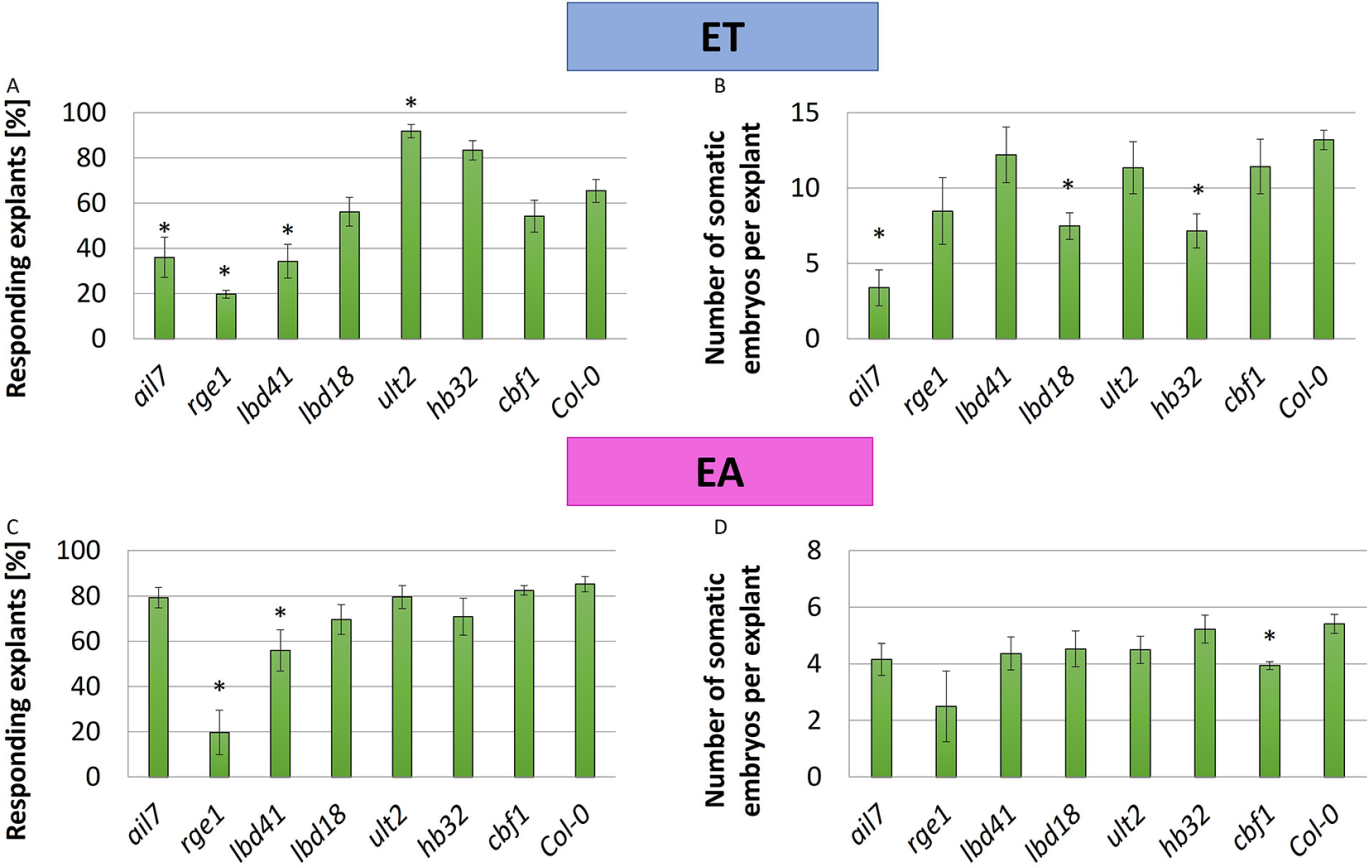
The embryogenic response of the polarity-related mutants (*ail7*, *rge1*, *lbd41*, *lbd18*, *ult2*, *hb32*, *cbf1*). SE efficiency (**A**, **C**) and SE productivity (**B**, **D**) of mutants and WT (Col-0) control cultures induced on the ET (**A**, **B**) and EA (**C**, **D**) medium for 21 days. Values significantly different from the control WT culture are marked with an asterisk (*n* = 3; means ± SD are given) (Student’s t-test, *p* < 0.05)

The role of organ polarity-related *TFs*, including *AINTEGUMENTA-LIKE 7/PLETHORA 7* (*AIL7/PLT7*), *RGE1*, *LOB DOMAIN-CONTAINING PROTEINs* (*LBD18*, *LBD41*), *HB32*, *CBF1*, *ULT2* in the SE induction was further explored, and the embryogenic response of the relevant mutants was evaluated (Fig. 10; Additional file17). The majority of the mutants (6/7) were found substantially affected in embryogenic response on ET medium, and most (5) of them, including *ail7*, *rge1*, *lbd18*, *lbd41*, and *hb32* manifested significantly reduced efficiency and/or productivity of SE. In EA culture, three mutants, including *rge1*, *lbd41*, and *cbf1* displayed impaired SE efficiency and/or SE productivity. In contrast to most of the analyzed mutants that were defective in embryogenic response, the *ult2* mutation showed improved SE efficiency in ET culture.

## Discussion

### Hac globally deregulates cell transcriptome in SE induction

The knowledge of how somatic cells can reprogram, differentiate, and regenerate plants via a plant-specific process of SE remains at the center of developmental biology research [84]. Studies on SE induction in Arabidopsis revealed the complex network of genes controlling embryogenic transition and pointed to the epigenetic mechanisms triggering the SE-involved genes that need to be revealed (reviewed in [10]) [85].

Here, we had insights into the TSA-induced embryogenic transcriptome of Arabidopsis culture to identify candidates of the Hac-regulated expression in SE. In line with the global increase of Hac and chromatin accessibility induced by TSA [35], we indicated the deregulation of a huge number of transcripts (44.4% out of 30,700) in the TSA-treated Arabidopsis explants. TSA-induced global deregulation of cell transcriptome was also associated with in vitro-induced cell reprogramming in plant protoplasts [36] and microspores of Arabidopsis [40]. Meaningfully, the global deregulation of genes was also associated with auxin-induced SE as indicated in present and other studies [86–88]. The similarity of TSA- and auxin-induced effects on plant cell transcriptome suggests that auxin and Hac play a prominent role in SE-related plant cell reprogramming. Moreover, the results imply that extensive changes in explant cell transcriptome might be an essential step of SE induction. In support of this, global transcriptome deregulation and stochastic gene expression have been postulated to play a common role in early events of cell fate reprogramming in plant protoplast culture [36]. In the proposed model, a stochastic gene expression pattern endows cells with heterogeneous fates, and out of these cells, sparse embryogenic cells are selected at a cellular level [36]. Also, in animals, stochasticity of gene deregulation contributed to reprogramming germ cells into somatic cells [89].

We indicated that an over-representation of downregulated transcripts, 56 and 61%, respectively, was the characteristic response of TSA and auxin-induced transcriptomes. Similarly, the excess of downregulated transcripts in response to TSA and auxin was also reported in other studies [56, 88, 90, 91]. In support of the role of gene repression in cell reprogramming including embryogenic transition, the essential function of gene repression in hormone signal transduction and response to stresses, the processes of the central role in SE induction, have been recognized [92]. Further studies on the contribution of global and gene-specific transcriptional repression in embryogenic reprogramming during SE induction are recommended.

### Candidate genes of Hac-regulated expression in SE induction

The RNA-seq results demonstrating the global deregulation of cell transcriptome in response to TSA treatment implied a substantial role of Hac in the reprogramming of cells during SE induction. To identify candidate genes of Hac-regulated expression in SE, we focused on TSA-deregulated transcripts encoding auxin-related genes due to the central role of auxin in embryogenic transition [93] and the involvement of Hac in auxin responses [94–96]. A positive effect of auxin treatment, including 2,4-D, on the Hac level in in vitro-cultured cells and tissue further evidenced the contribution of Hac to auxin-induced responses [97, 98]. In the current model of Hac-mediated regulation of auxin-responsive genes, auxin affects the recruitment of HATs and HDACs to the transcriptional complexes to differentially acetylate histones at the target *loci* (reviewed in [99]) [100]. In line with the model, the role of the HAG1 and HDA19 in controlling auxin-responsive *TFs*, *LEC1*, *LEC2*, and *BBM*, has been reported in SE [49].

Within SE-DEGs, a set of almost 3,400 genes similarly deregulated in response to both TSA and auxin might provide a valuable resource for identifying Hac-regulated genes of essential functions in auxin-induced SE. Genes of different functional groups were identified within the TSA and auxin-induced DEGs, and we get closer insights into transcripts related to auxin and stress of prominent regulatory role in SE induction [101, 102]. Moreover, within the Hac-regulated candidates, we identified a group of organ-polarity-related *TFs* that might have a role in SE induction.

### Genes involved in the signaling, biosynthesis, and polar transport of auxin

The availability of specific components of transcriptional complexes is essential for ensuring the correct regulation of genes, including those auxin-responsive [103, 104]. Accordingly, the accessibility of core components of auxin signaling, including AUX/IAAs and ARFs, might contribute to the regulation of auxin-responsive genes [103]. Thus, epigenetic processes controlling genes of auxin signaling components might control the transcription of auxin-regulated genes [105]. The reports on Hac-controlled expression of specific *ARF*s and *AUX/IAA* genes are limited and include the control of *ARF18* and *ARF22* by HDA710 during callus formation in rice [106] and *IAA3* regulation by HAT1/GCN5 in light response in Arabidopsis [107].

Thus, we searched for candidate *ARF* and *AUX/IAA* transcripts of Hac-regulated expression in SE. The RNA-seq results showed that most *AUX/IAA* and *ARF* genes were significantly deregulated in response to TSA implying the role of Hac in their transcriptional control in SE. TSA upregulated five *AUX/IAA (IAA1*, *20*, *29*, *30*, and *33*) and nine *ARF* (*ARF3*, *4*, *5*, *6*, *8*, *10*, *17*, *18*, *19*) genes, suggesting that HDAC might directly control these genes in SE induction. Within the candidate targets of HDAC, the *ARF5* encoding a central regulator of nuclear auxin signaling in plant development [108] was postulated to regulate different auxin-controlled aspects of SE, involving the tryptophan-dependent TAA1-YUC-mediated pathway of auxin biosynthesis (reviewed in [6]). In control of plant development, ARF5 may interact with IAA30, another candidate for Hac-mediated regulation (present results) of reported involvement in SE [109, 110]. Besides ARF5, also ARF10 and ARF16 might regulate the auxin biosynthesis in SE [111]. Also, *ARF3*, *ARF6*, and *ARF8* seem to control SE induction; however, the targeted processes need to be unveiled [112].

The RNA-seq results provided also other lines of suggestion for the role of Hac in auxin biosynthesis in SE. Accordingly, numerous DEGs encoding the main enzymes of Trp biosynthesis and the IPA pathway, such as aminotransferase TAA1 and YUC monooxygenases (reviewed in [99]), that contribute to auxin biosynthesis in auxin-induced SE [112] were found upregulated by TSA. Moreover, TSA induced an increase in Trp and IAA levels in SE. Together, the results imply the engagement of Hac in activating Trp- and IPA-biosynthetic pathways in SE induction. Relevantly, the role of HDA9 in the transcriptional activation of *YUC8* in the thermomorphogenesis of Arabidopsis was revealed [113]. Similar regulatory interaction might control SE induction since increased *HDA9* and *YUC8* expression was attributed to embryogenic induction (present results; [49, 114]. Other *YUC* genes, *YUC4* and *YUC10*, that were highly upregulated by TSA (present results), might also contribute to the YUC-dependent and LEC2-controlled auxin biosynthesis in SE [114].

In line with the increased expression of the genes encoding the YUC-pathway components in SE, we indicated the accumulation of IAA in the embryogenic cultures. Interestingly, the level of IAA was elevated in the advanced but not in the early stage of SE induced by both TSA and auxin. The result implies that similar to auxin-induced SE, induction of embryonic identity in explant tissue treated with TSA does not require *de novo* IAA biosynthesis in the early SE [115]. Moreover, the results suggest the similarity of auxin biosynthesis-related mechanisms controlling auxin and TSA-induced SE.

The results provide some hints that auxin biosynthesis pathways involved in SE might involve nitrilase-controlled pathways [79, 116, 117], including the significant modulation of *NIT* genes in embryogenic cultures, the distinctly impaired embryogenic response of *nit* mutants, and the downregulation of the *NIT* transcripts in the mutant of defective expression of *LEC1* TF, the main SE-regulator. Since *LEC1*, via controlling *LEC2*, might regulate the YUC-induced auxin biosynthesis in SE [118], we hypothesized that LEC1 might also control auxin biosynthesis in SE via an alternative NIT-involved pathway. Moreover, Hac might play a significant role in the regulation of NIT-dependent IAA biosynthesis in SE due to the highly intensive upregulation of nitrilase genes (*NIT1*, *2*, and *4*) in response to TSA (present results). The knowledge of epigenetic regulation of *NIT* genes is limited; however, changes in the Hac level in the promoter region of *NIT* genes were indicated in the PlantPAN3.0 database [119].

It is worth noting that NIT-mediated auxin biosynthesis has been reported to play roles in SE-related processes (present results), including stress responses and flowering of plants [120–122]. However, the contribution of the NIT pathway to auxin biosynthesis in plant development remains elusive [123]. The possible function of NITs in detoxifying ß-cyano alanine, an aside-product of ethylene biosynthesis, must also be considered while studying the role of NITs in SE induction [124]. In summary, the results raised questions about the involvement of the NIT-involved pathway of IAA biosynthesis in embryogenic induction and the role of Hac in the regulation of this alternative to the YUC-dependent route in SE.

In concert with biosynthesis, the polar transport of auxin and the major efflux and influx auxin carriers, relevantly PINs and AUX/LAXs, control embryonic transition and differentiation of pro-embryonic cells into somatic embryos [115, 125]. Accordingly, the present results showed that similar to auxin, TSA modulated the expression of numerous *PIN*, *AUX/LAX* genes, suggesting the role of Hac in regulating auxin carriers in SE. In support of this, the involvement of Hac and methylation in regulating *PIN1* in plant development was reported [126] (reviewed in [99]).

The candidate genes for Hac-regulated expression involve *PIN1*,*3* and *6* and *LAX1*, *2* of highly upregulated transcripts in ET and EA culture. PIN1 and PIN3 control the auxin gradient in zygotic embryogenesis (reviewed in [127]) and the role of PIN1 in regulating SE was evidenced [115, 125, 128]. The PIN1-mediated auxin movement establishes the *WUSCHEL*-expressing cells involved in somatic embryo formation [125]. In cooperation with PINs, auxin influx carriers such as AUX1 and redundantly acting LAX2 and LAX3, balance auxin levels and maintain embryonic cell identity in SE [115]. Further insights into the role of Hac in regulating the candidate auxin carriers (*PIN* and *AUX/LAX*) in SE are required.

### Stress-related genes

The role of stress factors in cell reprogramming, including the induction of SE in plants, has been widely demonstrated (reviewed in [129]). Particularly intensive upregulation of genes involved in biotic and abiotic stress responses [33, 130] in TSA-induced SE (present results) implies a prominent role of HDACs in regulating stress responses related to embryogenic induction. Consistent with this assumption, the involvement of *HDAC*s in controlling the expression of stress-related genes was reported [131–133].

Within candidate Hac-controlled and stress-related DEGs, we indicated the genes encoding oxidant and antioxidant enzymes. Also, other reports on TSA-induced transcriptomes support our assumption of Hac-mediated regulation of redox-controlling genes [56, 58]. In line with transcriptomic results, the increase of reactive oxygen (ROS) and nitrogen species was associated with SE induction and response to TSA [50, 134, 135]. ROS acting as signaling molecules in gene expression regulation might be accumulated as a result of increased SA production (reviewed in [136]). Consistently, we indicated the accumulation of SA accompanied by extensive deregulation of genes encoding critical enzymes in the metabolism of the stress phytohormones in the TSA-induced SE (present results). Besides ROS-mediated gene regulation, SA might also impact SE by contributing to the production of glutathione (GSH) [137] which promotes auxin biosynthesis in SE [135]. Besides SA, another stress-related phytohormone, ABA, that was accumulated in ET culture (present results) may also positively impact embryogenic induction by affecting gene expression and local auxin biosynthesis [138, 139].

We found that the *ICS1* gene involved in SA biosynthesis was upregulated in *hda19* mutants and response to TSA (present results) [140]. These results imply the HDA-mediated regulation of SA biosynthesis in SE induction. The candidate HDACs controlling SA-mediated stress responses in embryogenic induction involve HDA6 and HDA19 of indicated function in both SA biosynthesis and SE induction [49, 54, 140, 141].

The results pointed to the *NINE-CIS-EPOXYCAROTENOID DIOXYGENASE 6* (*NCED6*) as a candidate ABA biosynthesis gene that might be regulated by Hac in SE induction. The *NCED6* encodes one of the key stress-induced and rate-limiting dioxygenases in ABA biosynthesis (reviewed in [142]), and its transcripts were upregulated in TSA-induced culture. The role of Hac in the regulation of *NCED*-mediated ABA biosynthesis was reported in dehydration stress [143]. The candidate HDACs that might regulate ABA biosynthesis in SE include HDA19 and HDA15 which regulated *NCED* in seed dormancy and response to salt stress [144, 145]. The role of these enzymes in SE implies the differential expression of *HDA19* and *HDA15* genes in embryogenic cultures of Arabidopsis [49].

SA and ABA were accumulated specifically in TSA- but not auxin-induced cultures and that implied some differences in stress hormone-related embryogenic responses induced by auxin and TSA treatments. In contrast to SA and ABA, both treatments similarly modulated levels of JA in SE (present results). Regulatory interactions of JA and auxin in SE might be assumed, and JA positively affected IAA biosynthesis by upregulating *JASMONATE-ZIM-PROTEIN* (*JAZ1*) controlling IAA accumulation and SE induction in Arabidopsis [146].

Altogether, the results provided new evidence that Hac plays a key role in the epigenetic regulation of stress responses, including stress hormone-related metabolic pathways of central position in embryogenic induction (reviewed in [147]) [148]. The function and Hac-mediated regulatory mechanism controlling stress-related candidate genes that were identified in the present study need verification and further functional analysis.

### TFs regulators of organ polarity

The present and previous work showed that mostly the adaxial side of the IZE cotyledons was involved in auxin and TSA-induced SE of Arabidopsis [50]. Similarly, the cotyledon explant polarity affected SE induction in other plant species [86, 149, 150]. However, genetic and epigenetic regulators of polar embryogenic responses of the explants remain unidentified.

The cotyledon polarity is established early in zygotic embryogenesis and the expression of adaxial–abaxial identity genes was observed in globular-stage cotyledons [151, 152]. A higher expression of SE-*TFs*, including *LEC2* [153], *WOX2*, and *AT-HOOK MOTIF NUCLEAR LOCALIZED 15* (*AHL15*) genes [115] in the adaxial side of cotyledons implied the role of dorsoventral gene expression polarity in the mechanism controlling SE induction. Moreover, markers of the leaf adaxial surface, including *ARF5*, *PHB*, *PHV*, *REV*, *WOXs*, *ASYMMETRIC LEAVES* (*AS1*, *2*) genes [154, 155], were upregulated in the embryogenic culture (present results) [156]. Most of these markers, including *ARF5*, *PHB*, *PHV*, *REV*, and *WOXs*, were indicated to control SE [111, 112, 157]. The present SE-DEGs analysis revealed a novel group of organ polarity-related TFs, including *AIL7/PLT7*, *RGE1*, *LBD18*, *41*, *HB32*, *CBF1* and *ULT2* (Additional file16). Supportive for the role of these genes in SE induction, the relevant mutants were affected in SE response (present results). Moreover, TFs that are involved in both organ pattern formation and SE induction have auxin-related functions [158, 159], including the AIL7/PLT7, from the AIL family of PLETHORA (PLT) [160] and CBF1 [161]. CBF1 might contribute to SE by controlling auxin biosynthesis through PIF4 of PHYTOCHROME-INTERACTING FACTOR family of bHLH TFs that play a central role in modulating plant growth and development [161]. In support of this assumption, the regulatory relationship of PIF4 and the SE-involved YUC-mediated auxin biosynthesis pathway was reported [114, 162]. Interestingly, during low-temperature stress, *CBF1 TF* is regulated by a complex of ICE1 (INDUCER OF CBF EXPRESSION 1) and RGE1 TF, another SE-regulatory candidate of organ polarity DEGs [163]. In endosperm, RGE1 in complex with ICE1, represses an important ABA-regulator, the *ABI3* gene of the LAFL TFs group [164], of which members (LEC1, LEC2, ABI3, and FUS3) have master regulatory functions in SE (reviewed in [10]).

The ULT2, another organ-polarity-related TF of TSA-increased expression in SE, has overlapping functions with ULT1, which regulates auxin signaling in the apical root and floral meristem [165, 166]. We assumed that similarly to regulatory relationships in the floral meristem, ULTs may affect auxin signaling in SE by WUS TF of the central position in the SE regulatory network [167, 168]. Together with the increased embryogenic potential of *ult2* mutant (present results), these reports suggest that ULT2 might negatively regulate *WUS* and SE. The hypothesis needs validation.

In contrast to *ULT2*, mutant analysis of the *LBD18* and *LBD41* of the *LATERAL ORGAN BOUNDARIES* (*LOB*) domain *TF* gene family members implied the positive control of SE induction by these TFs (present results). LBD18 and LBD41 are involved in adaxial cell fate specialization [169] and the lateral root primordium developmental pathway [170], relevantly, the processes similar to in vitro-induced dedifferentiation of explant tissue [171, 172]. Moreover, in lateral root formation, LBD18 regulates *ARFs*, including *ARF19* of increased expression in auxin- and TSA-induced SE (present results) [173]. Thus, we assumed that targets of LBD18 may provide candidates in the search for auxin signaling pathway components controlling SE induction.

The particularly extensive upregulation of the organ-polarity *TFs* in response to TSA (present results) implied that Hac might control these genes in SE. The limited reports on the Hac role in the regulation of organ-polarity genes pointed to HDACs, HD2A, and HD2B, in establishing leaf polarity in the mutants in *ASYMMETRIC LEAVES* (*AS1*, *2*) [174]. In addition, acetylation of histone marks has been indicated in the chromatin associated with organ-polarity-related DEGs, including *AIL7/PLT7*, *RGE1*, *LBD18*, *LBD41*, *HB32*, *CBF1*, and *ULT2* (PlantPAN 3.0, [119]). Further studies may help to reveal the Hac-related mechanism controlling the embryogenic response of adaxial cotyledonary cells in Arabidopsis.

## Conclusions

The study indicated that Hac controls the vast majority of the genes in SE induction and evidenced that this epigenetic mark plays a pronounced function in the fine-tuning of plant somatic cell transcriptome during the embryogenic transition. Numerous candidates of Hac-regulated expression were identified, particularly the genes of auxin- and stress-related functions of an essential contribution in embryogenic responses. A novel group of organ-polarity genes was also identified and postulated to play a critical function in SE induction. The results provide a unique database for studies on the role of the Hac-related epigenetic regulation of embryogenic response that is induced in vitro in somatic plant cells.

### Electronic supplementary material

Below is the link to the electronic supplementary material.

Additional file 1: Table S1

Additional file 2: Fig. S1

Additional file 3: Fig. S2

Additional file 4: Fig. S3

Additional file 5: Dataset S1

Additional file 6: Dataset S2

Additional file 7: Dataset S3

Additional file 8: Fig. S4

Additional file 9: Dataset S4

Additional file 10: Table S2

Additional file 11: Dataset S5

Additional file 12: Dataset S6

Additional file 13: Table S3

Additional file 14: Table S4

Additional file 15: Fig. S5

Additional file 16: Table S5

Additional file 17: Fig. S6

Additional file 18: Legends

## Data Availability

The RNA-seq data presented in this publication have been deposited in NCBI’s Gene Expression Omnibus (GEO) and are accessible through GEO Series accession number GSE255229. Data generated or analysed during this study are included in this published article. Data is provided within the manuscript or supplementary information files. Any additional information is available from the corresponding author on reasonable request.

## Abbreviations

2,4-D: 2,4-Dichlorophenoxyacetic acid
ABA: Abscisic acid
DEGs: Differentially expressed genes
E0: Auxin and trichostatin A free medium
EA: Embryogenesis induction medium with 5 µM 2,4-D
ET: Embryogenesis induction medium with 1 µM trichostatin A
Hac: Histone acetylation
IAA: Indole-3-acetic acid
IZE: Immature zygotic embryo
JA: Jasmonic acid
SA: Salicylic acid
SE: Somatic embryogenesis
TF: Transcription factor
Trp: Tryptophan
TSA: Trichostatin A
GO: Gene ontology

## References

[1] Garcês HM, Koenig D, Townsley BT, Kim M, Sinha NR. Truncation of LEAFY COTYLEDON1 protein is required for asexual reproduction in *Kalanchoë daigremontiana*. Plant Physiol. 2014;165(1):196–206. 24664206 10.1104/pp.114.237222 PMC4012580

[2] Elhiti M, Stasolla C. The use of zygotic embryos as explants for in vitro propagation: an overview. Methods Mol Biol. 2011;710:229–55. 21207273 10.1007/978-1-61737-988-8_17

[3] Méndez-Hernández HA, Ledezma-Rodríguez M, Avilez-Montalvo RN, Juárez-Gómez YL, Skeete A, Avilez-Montalvo J. De-la-Peña C, Loyola-Vargas VM: signaling overview of plant somatic embryogenesis. Front Plant Sci. 2019;10:77. 30792725 10.3389/fpls.2019.00077 PMC6375091

[4] Ochoa-Alejo N. The uses of somatic embryogenesis for genetic transformation. In: Somatic Embryogenesis: Fundamental Aspects And Applications Edited by Loyola-Vargas V, Ochoa-Alejo, N. Cham, Switzerland: Springer International Publishing; 2016: 415–434.

[5] Egertsdotter U, Ahmad I, Clapham D. Automation and scale up of somatic embryogenesis for commercial plant production, with emphasis on conifers. Front Plant Sci. 2019;10:109. 30833951 10.3389/fpls.2019.00109 PMC6388443

[6] Wójcik AM, Wójcikowska B, Gaj MD. Current perspectives on the auxin-mediated genetic network that controls the induction of somatic embryogenesis in plants. Int J Mol Sci. 2020;21(4):1333. 32079138 10.3390/ijms21041333 PMC7072907

[7] Hofmann F, Schon MA, Nodine MD. The embryonic transcriptome of Arabidopsis thaliana. Plant Reprod. 2019;32(1):77–91. 30610360 10.1007/s00497-018-00357-2

[8] Ibáñez S, Carneros E, Testillano PS, Pérez-Pérez JM. Advances in plant regeneration: shake, rattle and roll. Plants (Basel). 2020;9(7):897. 32708602 10.3390/plants9070897 PMC7412315

[9] Wójcikowska B, Wójcik AM, Gaj MD. Epigenetic regulation of auxin-induced somatic embryogenesis in plants. Int J Mol Sci. 2020;21(7):2307. 32225116 10.3390/ijms21072307 PMC7177879

[10] Salaün C, Lepiniec L, Dubreucq B. Genetic and molecular control of somatic embryogenesis. Plants (Basel). 2021;10(7):1467. 34371670 10.3390/plants10071467 PMC8309254

[11] Gulzar B, Mujib A, Malik MQ, Sayeed R, Mamgain J, Ejaz B. Genes, proteins and other networks regulating somatic embryogenesis in plants. J Genet Eng Biotechnol. 2020;18(1):31. 32661633 10.1186/s43141-020-00047-5 PMC7359197

[12] De-la-Peña C, Nic-Can GI, Galaz-Ávalos RM, Avilez-Montalvo R, Loyola-Vargas VM. The role of chromatin modifications in somatic embryogenesis in plants. Front Plant Sci. 2015;6:635. 26347757 10.3389/fpls.2015.00635 PMC4539545

[13] Birnbaum KD, Roudier F. Epigenetic memory and cell fate reprogramming in plants. Regeneration (Oxf). 2017;4(1):15–20. 28316791 10.1002/reg2.73 PMC5350078

[14] Nic-Can GI, López-Torres A, Barredo-Pool F, Wrobel K, Loyola-Vargas VM, Rojas-Herrera R, De-la-Peña C. New insights into somatic embryogenesis: leafy cotyledon1, baby boom1 and WUSCHEL-related homeobox4 are epigenetically regulated in Coffea canephora. PLoS ONE. 2013;8(8):e72160. 23977240 10.1371/journal.pone.0072160 PMC3748027

[15] Karim R, Tan YS, Singh P, Khalid N, Harikrishna JA. Expression and DNA methylation of SERK, BBM, LEC2 and WUS genes in in vitro cultures of Boesenbergia rotunda (L.) Mansf. Physiol Mol Biol Plants. 2018;24(5):741–51. 30150851 10.1007/s12298-018-0566-8 PMC6103949

[16] Grzybkowska D, Morończyk J, Wójcikowska B, Gaj MD. Azacitidine (5-AzaC)-treatment and mutations in DNA methylase genes affect embryogenic response and expression of the genes that are involved in somatic embryogenesis in Arabidopsis. Plant Growth Regul. 2018;85(2):243–56. 10.1007/s10725-018-0389-1

[17] Grzybkowska D, Nowak K, Gaj MD. Hypermethylation of auxin-responsive motifs in the promoters of the transcription factor genes accompanies the somatic embryogenesis induction in Arabidopsis. Int J Mol Sci. 2020;21(18):6849. 32961931 10.3390/ijms21186849 PMC7555384

[18] Li J, Wang M, Li Y, Zhang Q, Lindsey K, Daniell H, Jin S, Zhang X. Multi-omics analyses reveal epigenomics basis for cotton somatic embryogenesis through successive regeneration acclimation process. Plant Biotechnol J. 2019;17(2):435–50. 29999579 10.1111/pbi.12988 PMC6335067

[19] Grafi G, Ben-Meir H, Avivi Y, Moshe M, Dahan Y, Zemach A. Histone methylation controls telomerase-independent telomere lengthening in cells undergoing dedifferentiation. Dev Biol. 2007;306(2):838–46. 17448460 10.1016/j.ydbio.2007.03.023

[20] Bouyer D, Roudier F, Heese M, Andersen ED, Gey D, Nowack MK, Goodrich J, Renou JP, Grini PE, Colot V, et al. Polycomb repressive complex 2 controls the embryo-to-seedling phase transition. PLoS Genet. 2011;7(3):e1002014. 21423668 10.1371/journal.pgen.1002014 PMC3053347

[21] Chen D, Molitor A, Liu C, Shen WH. The Arabidopsis PRC1-like ring-finger proteins are necessary for repression of embryonic traits during vegetative growth. Cell Res. 2010;20(12):1332–44. 21060339 10.1038/cr.2010.151

[22] Rose RJ. Somatic embryogenesis in the *Medicago truncatula* model: cellular and molecular mechanisms. Front Plant Sci. 2019;10:267. 30984208 10.3389/fpls.2019.00267 PMC6447896

[23] Mozgová I, Muñoz-Viana R, Hennig L. PRC2 represses hormone-Induced somatic embryogenesis in vegetative tissue of Arabidopsis thaliana. PLoS Genet. 2017;13(1):e1006562. 28095419 10.1371/journal.pgen.1006562 PMC5283764

[24] Liang Z, Riaz A, Chachar S, Ding Y, Du H, Gu X. Epigenetic modifications of mRNA and DNA in plants. Mol Plant. 2020;13(1):14–30. 31863849 10.1016/j.molp.2019.12.007

[25] Zhao T, Zhan Z, Jiang D. Histone modifications and their regulatory roles in plant development and environmental memory. J Genet Genomics. 2019;46(10):467–76. 31813758 10.1016/j.jgg.2019.09.005

[26] Lee CY, Grant PA. Role of histone acetylation and acetyltransferases in gene regulation. In: *Toxicoepigenetics* Edited by McCullough S, Dolinoy, D. Cambridge, MA, USA: Elsevier Academic Press; 2019: 3–30.

[27] Carlberg C, Molnár F. Histone modifications. In: Human epigenetics: How science works Edited by Carlberg C, Molnár F. Cham, Switzerland Springer International Publishing; 2019: 41–50.

[28] Shen Y, Wei W, Zhou DX. Histone acetylation enzymes coordinate metabolism and gene expression. Trends Plant Sci. 2015;20(10):614–21. 26440431 10.1016/j.tplants.2015.07.005

[29] Roche J, Bertrand P. Inside HDACs with more selective HDAC inhibitors. Eur J Med Chem. 2016;121:451–83. 27318122 10.1016/j.ejmech.2016.05.047

[30] Trisciuoglio D, Rotili D. Histone acetyltransferase enzymes: from biological implications to most relevant inhibitors. In: Chemical Epigenetics Edited by Mai A. Cham, Switzerland: Springer International Publishing; 2019: 93–122.

[31] Elvir L, Duclot F, Wang Z, Kabbaj M. Epigenetic regulation of motivated behaviors by histone deacetylase inhibitors. Neurosci Biobehav Rev. 2019;105:305–17. 29020607 10.1016/j.neubiorev.2017.09.030 PMC5889966

[32] Finnin MS, Donigian JR, Cohen A, Richon VM, Rifkind RA, Marks PA, Breslow R, Pavletich NP. Structures of a histone deacetylase homologue bound to the TSA and SAHA inhibitors. Nature. 1999;401(6749):188–93. 10490031 10.1038/43710

[33] Venturelli S, Belz RG, Kämper A, Berger A, von Horn K, Wegner A, Böcker A, Zabulon G, Langenecker T, Kohlbacher O, et al. Plants release precursors of histone deacetylase inhibitors to suppress growth of competitors. Plant Cell. 2015;27(11):3175–89. 26530086 10.1105/tpc.15.00585 PMC4682303

[34] Mengel A, Ageeva A, Georgii E, Bernhardt J, Wu K, Durner J, Lindermayr C. Nitric oxide modulates histone acetylation at stress genes by inhibition of histone deacetylases. Plant Physiol. 2017;173(2):1434–52. 27980017 10.1104/pp.16.01734 PMC5291017

[35] Görisch SM, Wachsmuth M, Tóth KF, Lichter P, Rippe K. Histone acetylation increases chromatin accessibility. J Cell Sci. 2005;118(Pt 24):5825–34. 16317046 10.1242/jcs.02689

[36] Xu M, Du Q, Tian C, Wang Y, Jiao Y. Stochastic gene expression drives mesophyll protoplast regeneration. Sci Adv. 2021;7(33):eabg8466. 34380624 10.1126/sciadv.abg8466 PMC8357238

[37] Qiu X, You H, Xiao X, Li N, Li Y. Effects of Trichostatin A and PXD101 on the in vitro development of mouse somatic cell nuclear transfer embryos. Cell Reprogram. 2017;19(1):1–9. 28112984 10.1089/cell.2016.0030

[38] Al-Ghadi MQ, Alhimaidi AR, Iwamoto D, Al-Mutary MG, Ammari AA, Saeki KO, Aleissa MS. The *in vitro* development of cloned sheep embryos treated with scriptaid and trichostatin (A). Saudi J Biol Sci. 2020;27(9):2280–6. 32884408 10.1016/j.sjbs.2020.04.039 PMC7451688

[39] Liu Z, Cai Y, Wang Y, Nie Y, Zhang C, Xu Y, Zhang X, Lu Y, Wang Z, Poo M, et al. Cloning of macaque monkeys by somatic cell nuclear transfer. Cell. 2018;172(4):881–7. 29395327 10.1016/j.cell.2018.01.020

[40] Li H, Soriano M, Cordewener J, Muiño JM, Riksen T, Fukuoka H, Angenent GC, Boutilier K. The histone deacetylase inhibitor trichostatin a promotes totipotency in the male gametophyte. Plant Cell. 2014;26(1):195–209. 24464291 10.1105/tpc.113.116491 PMC3963568

[41] Boutilier K, Angenent GC, Soriano Castan M. L H: Haploid embryogenesis. In., vol. WO 2015/043621; 2015.

[42] Castillo AM, Valero-Rubira I, Burrell MÁ, Allué S, Costar MA, Vallés MP. Trichostatin A affects developmental reprogramming of bread wheat microspores towards an embryogenic route. Plants (Basel). 2020;9(11):1442. 33114625 10.3390/plants9111442 PMC7693754

[43] Jiang F, Ryabova D, Diedhiou J, Hucl P, Randhawa H, Marillia EF, Foroud NA, Eudes F, Kathiria P. Trichostatin A increases embryo and green plant regeneration in wheat. Plant Cell Rep. 2017;36(11):1701–6. 28752355 10.1007/s00299-017-2183-3

[44] Martínez Ó, Arjones V, González MV, Rey M. Histone deacetylase inhibitors increase the embryogenic potential and alter the expression of embryogenesis-related and HDAC-encoding genes in grapevine (*Vitis vinifera* L., Cv. Mencia). Plants (Basel). 2021;10(6):1164. 34201224 10.3390/plants10061164 PMC8228518

[45] Uddenberg D, Valladares S, Abrahamsson M, Sundström JF, Sundås-Larsson A, von Arnold S. Embryogenic potential and expression of embryogenesis-related genes in conifers are affected by treatment with a histone deacetylase inhibitor. Planta. 2011;234(3):527–39. 21541665 10.1007/s00425-011-1418-8 PMC3162143

[46] Abrahamsson M, Valladares S, Merino I, Larsson E, von Arnold S. Degeneration pattern in somatic embryos of *Pinus sylvestris* L. Vitro Cell Dev Biol Plant. 2017;53(2):86–96. 10.1007/s11627-016-9797-y PMC5423931 28553064

[47] Awada R, Verdier D, Froger S, Brulard E, de Faria Maraschin S, Etienne H, Breton D. An innovative automated active compound screening system allows high-throughput optimization of somatic embryogenesis in Coffea arabica. Sci Rep. 2020;10(1):810. 31965007 10.1038/s41598-020-57800-6 PMC6972844

[48] Rodríguez-Sanz H, Moreno-Romero J, Solís M-T, Köhler C, Risueño MC, Testillano PS. Changes in histone methylation and acetylation during microspore reprogramming to embryogenesis occur concomitantly with Bn*HKMT* and Bn*HAT* expression and are associated with cell totipotency, proliferation, and differentiation in *Brassica napus*. Cytogenet Genome Res. 2014;143(1–3):209–18. 25060767 10.1159/000365261

[49] Morończyk J, Brąszewska A, Wójcikowska B, Chwiałkowska K, Nowak K, Wójcik AM, Kwaśniewski M, Gaj MD. Insights into the histone acetylation-mediated regulation of the transcription factor genes that control the embryogenic transition in the somatic cells of Arabidopsis. Cells. 2022;11(5):863. 35269485 10.3390/cells11050863 PMC8909028

[50] Wójcikowska B, Botor M, Morończyk J, Wójcik AM, Nodzyński T, Karcz J, Gaj MD. Trichostatin a triggers an embryogenic transition in Arabidopsis explants via an auxin-related pathway. Front Plant Sci. 2018;9:1353. 30271420 10.3389/fpls.2018.01353 PMC6146766

[51] Zhou Y, Tan B, Luo M, Li Y, Liu C, Chen C, Yu C-W, Yang S, Dong S, Ruan J. HISTONE DEACETYLASE19 interacts with HSL1 and participates in the repression of seed maturation genes in Arabidopsis seedlings. Plant Cell. 2013;25(1):134–48. 23362207 10.1105/tpc.112.096313 PMC3584530

[52] Ryu H, Cho H, Bae W, Hwang I. Control of early seedling development by BES1/TPL/HDA19-mediated epigenetic regulation of *ABI3*. Nat Commun. 2014;5:4138. 24938150 10.1038/ncomms5138

[53] Chhun T, Chong SY, Park BS, Wong EC, Yin JL, Kim M, Chua NH. HSI2 repressor recruits MED13 and HDA6 to down-regulate seed maturation gene expression directly during Arabidopsis early seedling growth. Plant Cell Physiol. 2016;57(8):1689–706. 27335347 10.1093/pcp/pcw095

[54] Nowak K, Morończyk J, Wójcik A, Gaj MD. AGL15 controls the embryogenic reprogramming of somatic cells in Arabidopsis through the histone acetylation-mediated repression of the miRNA biogenesis genes. Int J Mol Sci. 2020;21(18):6733. 32937992 10.3390/ijms21186733 PMC7554740

[55] Nowak K, Morończyk J, Grzyb M, Szczygieł-Sommer A, Gaj MD. miR172 regulates WUS during somatic embryogenesis in Arabidopsis via AP2. Cells. 2022;11(4):718. 10.3390/cells11040718 PMC8869827 35203367

[56] Ma X, Zhang C, Zhang B, Yang C, Li S. Identification of genes regulated by histone acetylation during root development in *Populus trichocarpa*. BMC Genom. 2016;17:96. 10.1186/s12864-016-2407-x PMC4743431 26847576

[57] Luo M, Tai R, Yu CW, Yang S, Chen CY, Lin WD, Schmidt W, Wu K. Regulation of flowering time by the histone deacetylase HDA5 in Arabidopsis. Plant J. 2015;82(6):925–36. 25922987 10.1111/tpj.12868

[58] Song Y, Liu L, Li G, An L, Tian L. Trichostatin A and 5-Aza-2’-Deoxycytidine influence the expression of cold-induced genes in Arabidopsis. Plant Signal Behav. 2017;12(11):e1389828. 29027833 10.1080/15592324.2017.1389828 PMC5703259

[59] Gaj MD. Direct somatic embryogenesis as a rapid and efficient system for *in vitro* regeneration of *Arabidopsis thaliana*. Plant Cell Tissue Organ Cult. 2001;64(1):39–46. 10.1023/A:1010679614721

[60] Gamborg OLc, Miller RA, Ojima K. Nutrient requirements of suspension cultures of soybean root cells. Exp Cell Res. 1968;50(1):151–8. 5650857 10.1016/0014-4827(68)90403-5

[61] Andrews S. FastQC: a quality control tool for high throughput sequence data [Online]. Available online at: http://www.bioinformatics.babraham.ac.uk/projects/fastqc/. 2010.

[62] Ewels P, Magnusson M, Lundin S, Kaller M. MultiQC: summarize analysis results for multiple tools and samples in a single report. Bioinformatics. 2016;32(19):3047–8. 27312411 10.1093/bioinformatics/btw354 PMC5039924

[63] Joshi N, Fass J. Sickle: a sliding-window, adaptive, quality-based trimming tool for FastQ files (Version 1.33)[Software]. In.; 2011.

[64] Kopylova E, Noé L, Touzet H. SortMeRNA: fast and accurate filtering of ribosomal RNAs in metatranscriptomic data. Bioinformatics. 2012;28(24):3211–7. 23071270 10.1093/bioinformatics/bts611

[65] Dobin A, Davis CA, Schlesinger F, Drenkow J, Zaleski C, Jha S, Batut P, Chaisson M, Gingeras TR. STAR: ultrafast universal RNA-seq aligner. Bioinformatics. 2013;29(1):15–21. 23104886 10.1093/bioinformatics/bts635 PMC3530905

[66] Lassmann T, Hayashizaki Y, Daub CO. SAMStat: monitoring biases in next generation sequencing data. Bioinformatics. 2011;27(1):130–1. 21088025 10.1093/bioinformatics/btq614 PMC3008642

[67] Okonechnikov K, Conesa A, García-Alcalde F. Qualimap 2: advanced multi-sample quality control for high-throughput sequencing data. Bioinformatics. 2016;32(2):292–4. 26428292 10.1093/bioinformatics/btv566 PMC4708105

[68] Li H, Handsaker B, Wysoker A, Fennell T, Ruan J, Homer N, Marth G, Abecasis G, Durbin R. Genome Project Data Processing S: the sequence alignment/map format and SAMtools. Bioinformatics. 2009;25(16):2078–9. 19505943 10.1093/bioinformatics/btp352 PMC2723002

[69] Thorvaldsdóttir H, Robinson JT, Mesirov JP. Integrative Genomics Viewer (IGV): high-performance genomics data visualization and exploration. Brief Bioinform. 2013;14(2):178–92. 22517427 10.1093/bib/bbs017 PMC3603213

[70] Love MI, Huber W, Anders S. Moderated estimation of Fold change and dispersion for RNA-seq data with DESeq2. Genome Biol. 2014;15(12):550. 25516281 10.1186/s13059-014-0550-8 PMC4302049

[71] Benjamini Y, Hochberg Y. Controlling the false discovery rate: a practical and powerful approach to multiple testing. J R Stat Soc Ser B Stat Methodol. 1995;57(1):289–300. 10.1111/j.2517-6161.1995.tb02031.x

[72] Zhou Y, Zhou B, Pache L, Chang M, Khodabakhshi AH, Tanaseichuk O, et al. Metascape provides a biologist-oriented resource for the analysis of systems-level datasets. Nat Commun. 2019;10(1):1523. 10.1038/s41467-019-09234-6 PMC6447622 30944313

[73] Ge SX, Jung D, Yao R. ShinyGO: a graphical gene-set enrichment tool for animals and plants. Bioinformatics. 2020;36(8):2628–29. 10.1093/bioinformatics/btz931 PMC7178415 31882993

[74] Le Roux C, Del Prete S, Boutet-Mercey S, Perreau F, Balague C, Roby D, Fagard M, Gaudin V. The hnRNP-Q protein LIF2 participates in the plant immune response. PLoS ONE. 2014;9(6):e99343. 24914891 10.1371/journal.pone.0099343 PMC4051675

[75] Jain A, Jain R, Jain S. Thin layer chromatography of amino acid. In: Basic Techniques in Biochemistry, Microbiology and Molecular Biology: Principles and Techniques Edited by Jain A, Jain R, Jain S. New York, NY: Springer US; 2020: 255–257.

[76] Csupor D, Boros K, Hunyadi A, Veres K, Hohmann J. Validation of a densitometric method for the determination of theanine in tea extracts using CP atlas software. JPC-Journal Planar Chromatography-Modern TLC. 2012;25(6):571–4. 10.1556/JPC.25.2012.6.14

[77] Doyle JJ, Doyle JL. A rapid DNA isolation procedure for small quantities of fresh leaf tissue. Phytochem Bull. 1987;19:11–5.

[78] Thellin O, Zorzi W, Lakaye B, De Borman B, Coumans B, Hennen G, Grisar T, Igout A, Heinen E. Housekeeping genes as internal standards: use and limits. J Biotechnol. 1999;75(2–3):291–5. 10617337 10.1016/S0168-1656(99)00163-7

[79] Bartling D, Seedorf M, Mithöfer A, Weiler EW. Cloning and expression of an Arabidopsis nitrilase which can convert indole-3-acetonitrile to the plant hormone, indole-3-acetic acid. Eur J Biochem. 1992;205(1):417–24. 1555601 10.1111/j.1432-1033.1992.tb16795.x

[80] Vorwerk S, Biernacki S, Hillebrand H, Janzik I, Müller A, Weiler EW, Piotrowski M. Enzymatic characterization of the recombinant *Arabidopsis thaliana* nitrilase subfamily encoded by the *NIT 2*/*NIT 1*/*NIT 3*-gene cluster. Planta. 2001;212:508–16. 11525507 10.1007/s004250000420

[81] Pelletier JM, Kwong RW, Park S, Le BH, Baden R, Cagliari A, Hashimoto M, Munoz MD, Fischer RL, Goldberg RB. LEC1 sequentially regulates the transcription of genes involved in diverse developmental processes during seed development. Proc Natl Acad Sci U S A. 2017;114(32):E6710–9. 28739919 10.1073/pnas.1707957114 PMC5559047

[82] Junker A, Mönke G, Rutten T, Keilwagen J, Seifert M, Thi TMN, Renou JP, Balzergue S, Viehöver P, Hähnel U. Elongation-related functions of LEAFY COTYLEDON1 during the development of *Arabidopsis thaliana*. Plant J. 2012;71(3):427–42. 22429691 10.1111/j.1365-313X.2012.04999.x

[83] Fehér A. Somatic embryogenesis - stress-induced remodeling of plant cell fate. Biochim Biophys Acta. 2015;1849(4):385–402. 25038583 10.1016/j.bbagrm.2014.07.005

[84] Sivanesan I, Nayeem S, Venkidasamy B, Kuppuraj SP, Rn C, Samynathan R. Genetic and epigenetic modes of the regulation of somatic embryogenesis: a review. Biol Futur. 2022;73(3):259–77. 35829936 10.1007/s42977-022-00126-3

[85] Kumar V, Van Staden J. New insights into plant somatic embryogenesis: an epigenetic view. Acta Physiol Plant. 2017;39(9):194. 10.1007/s11738-017-2487-5

[86] Thibaud-Nissen F, Shealy RT, Khanna A, Vodkin LO. Clustering of microarray data reveals transcript patterns associated with somatic embryogenesis in soybean. Plant Physiol. 2003;132(1):118–36. 12746518 10.1104/pp.103.019968 PMC166958

[87] Sharma SK, Millam S, Hedley PE, McNicol J, Bryan GJ. Molecular regulation of somatic embryogenesis in potato: an auxin led perspective. Plant Mol Biol. 2008;68(1–2):185–201. 18553172 10.1007/s11103-008-9360-2

[88] Wickramasuriya AM, Dunwell JM. Global scale transcriptome analysis of Arabidopsis embryogenesis *in vitro*. BMC Genom. 2015;16:301. 10.1186/s12864-015-1504-6 PMC4404573 25887996

[89] Robert VJ, Garvis S, Palladino F. Repression of somatic cell fate in the germline. Cell Mol Life Sci. 2015;72(19):3599–620. 26043973 10.1007/s00018-015-1942-y PMC11113910

[90] Chang S, Pikaard CS. Transcript profiling in Arabidopsis reveals complex responses to global inhibition of DNA methylation and histone deacetylation. J Biol Chem. 2005;280(1):796–804. 15516340 10.1074/jbc.M409053200

[91] Ogita S, Nomura T, Kato Y, Uehara-Yamaguchi Y, Inoue K, Yoshida T, Sakurai T, Shinozaki K, Mochida K. Transcriptional alterations during proliferation and lignification in Phyllostachys nigra cells. Sci Rep. 2018;8(1):11347. 30054534 10.1038/s41598-018-29645-7 PMC6063902

[92] Krogan NT, Long JA. Why so repressed? Turning off transcription during plant growth and development. Curr Opin Plant Biol. 2009;12(5):628–36. 19700365 10.1016/j.pbi.2009.07.011 PMC2757442

[93] Pierroz G. Making babies: how auxin regulates somatic embryogenesis in Arabidopsis tissue culture. Plant J. 2023;113(1):5–6. 36585767 10.1111/tpj.16062

[94] Anzola JM, Sieberer T, Ortbauer M, Butt H, Korbei B, Weinhofer I, Müllner AE, Luschnig C. Putative Arabidopsis transcriptional adaptor protein (PROPORZ1) is required to modulate histone acetylation in response to auxin. Proc Natl Acad Sci U S A. 2010;107(22):10308–13. 20479223 10.1073/pnas.0913918107 PMC2890463

[95] Weiste C, Dröge-Laser W. The Arabidopsis transcription factor bZIP11 activates auxin-mediated transcription by recruiting the histone acetylation machinery. Nat Commun. 2014;5:3883. 24861440 10.1038/ncomms4883

[96] Kuhn A, Ramans Harborough S, McLaughlin HM, Natarajan B, Verstraeten I, Friml J, Kepinski S, Ostergaard L. Direct ETTIN-auxin interaction controls chromatin states in gynoecium development. Elife. 2020;9:e51787. 32267233 10.7554/eLife.51787 PMC7164952

[97] Law RD, Suttle JC. Chromatin remodeling in plant cell culture: patterns of DNA methylation and histone H3 and H4 acetylation vary during growth of asynchronous potato cell suspensions. Plant Physiol Biochem. 2005;43(6):527–34. 15922608 10.1016/j.plaphy.2005.03.014

[98] Rodríguez JL, Valledor L, Hasbún R, Sánchez P, Rodríguez R, Cañal MJ. The effects of hormone treatment on epigenetic marks during organogenesis in *Pinus radiata* D. Don embryos. J Plant Growth Regul. 2016;35(1):97–108. 10.1007/s00344-015-9510-0

[99] Mateo-Bonmatí E, Casanova-Sáez R, Ljung K. Epigenetic regulation of auxin homeostasis. Biomolecules. 2019;9:623. 31635281 10.3390/biom9100623 PMC6843323

[100] Nguyen CT, Tran GB, Nguyen NH. Homeostasis of histone acetylation is critical for auxin signaling and root morphogenesis. Plant Mol Biol. 2020;103(1–2):1–7. 32088831 10.1007/s11103-020-00985-1

[101] Salvo SA, Hirsch CN, Buell CR, Kaeppler SM, Kaeppler HF. Whole transcriptome profiling of maize during early somatic embryogenesis reveals altered expression of stress factors and embryogenesis-related genes. PLoS ONE. 2014;9(10):e111407. 25356773 10.1371/journal.pone.0111407 PMC4214754

[102] Wang Y, Li H-L, Zhou Y-K, Guo D, Zhu J-H, Peng S-Q. Transcriptomes analysis reveals novel insight into the molecular mechanisms of somatic embryogenesis in *Hevea brasiliensis*. BMC Genom. 2021;22(1):1–18. 10.1186/s12864-021-07501-9 PMC7953812 33711923

[103] Weijers D, Benkova E, Jäger KE, Schlereth A, Hamann T, Kientz M, Wilmoth JC, Reed JW, Jürgens G. Developmental specificity of auxin response by pairs of ARF and Aux/IAA transcriptional regulators. Embo j. 2005;24(10):1874–85. 15889151 10.1038/sj.emboj.7600659 PMC1142592

[104] Caumon H, Vernoux T. A matter of time: auxin signaling dynamics and the regulation of auxin responses during plant development. J Exp Bot. 2023;74(14):3887–902. 37042516 10.1093/jxb/erad132

[105] Luo J, Zhou JJ, Zhang JZ. *Aux/IAA* gene family in plants: molecular structure, regulation, and function. Int J Mol Sci. 2018;19(1):259. 29337875 10.3390/ijms19010259 PMC5796205

[106] Zhang H, Guo F, Qi P, Huang Y, Xie Y, Xu L, Han N, Xu L, Bian H. OsHDA710-mediated histone deacetylation regulates callus formation of rice mature embryo. Plant Cell Physiol. 2020;61(9):1646–60. 32592489 10.1093/pcp/pcaa086

[107] Benhamed M, Bertrand C, Servet C, Zhou DX, Arabidopsis. *GCN5*, *HD1*, and *TAF1*/*HAF2* interact to regulate histone acetylation required for light-responsive gene expression. *Plant Cell* 2006, 18(11):2893–2903. 10.1105/tpc.106.043489 PMC1693931 17085686

[108] Kou X, Zhao X, Wu B, Wang C, Wu C, Yang S, Zhou J, Xue Z. Auxin response factors are ubiquitous in plant growth and development, and involved in crosstalk between plant hormones: a review. Appl Sci. 2022;12(3):1360. 10.3390/app12031360

[109] Braybrook SA, Stone SL, Park S, Bui AQ, Le BH, Fischer RL, Goldberg RB, Harada JJ. Genes directly regulated by LEAFY COTYLEDON2 provide insight into the control of embryo maturation and somatic embryogenesis. Proc Natl Acad Sci U S A. 2006;103(9):3468–73. 16492731 10.1073/pnas.0511331103 PMC1413938

[110] Krogan NT, Yin X, Ckurshumova W, Berleth T. Distinct subclades of Aux/IAA genes are direct targets of ARF5/MP transcriptional regulation. New Phytol. 2014;204(3):474–83. 25145395 10.1111/nph.12994

[111] Wójcik AM, Nodine MD, Gaj MD. miR160 and miR166/165 contribute to the *LEC2*-mediated auxin response involved in the somatic embryogenesis induction in Arabidopsis. *Front Plant Sci* 2017, 8:2024. 10.3389/fpls.2017.02024 PMC5732185 29321785

[112] Wójcikowska B, Gaj MD. Expression profiling of *AUXIN RESPONSE FACTOR* genes during somatic embryogenesis induction in Arabidopsis. Plant Cell Rep. 2017;36(6):843–58. 28255787 10.1007/s00299-017-2114-3 PMC5486788

[113] van der Woude LC, Perrella G, Snoek BL, van Hoogdalem M, Novák O, van Verk MC, van Kooten HN, Zorn LE, Tonckens R, Dongus JA, et al. HISTONE DEACETYLASE 9 stimulates auxin-dependent thermomorphogenesis in Arabidopsis thaliana by mediating H2A.Z depletion. Proc Natl Acad Sci U S A. 2019;116(50):25343–54. 31767749 10.1073/pnas.1911694116 PMC6911240

[114] Wójcikowska B, Jaskóła K, Gąsiorek P, Meus M, Nowak K, Gaj MD. *LEAFY COTYLEDON2* (*LEC2*) promotes embryogenic induction in somatic tissues of Arabidopsis, via *YUCCA*-mediated auxin biosynthesis. Planta. 2013;238(3):425–40. 23722561 10.1007/s00425-013-1892-2 PMC3751287

[115] Karami O, Philipsen C, Rahimi A, Nurillah AR, Boutilier K, Offringa R. Endogenous auxin maintains embryonic cell identity and promotes somatic embryo development in Arabidopsis. Plant J. 2023;113(1):7–22. 36345646 10.1111/tpj.16024 PMC10098609

[116] Normanly J, Grisafi P, Fink GR, Bartel B. Arabidopsis mutants resistant to the auxin effects of indole-3-acetonitrile are defective in the nitrilase encoded by the *NIT1* gene. Plant Cell. 1997;9(10):1781–90. 9368415 10.1105/tpc.9.10.1781 PMC157021

[117] Park WJ, Kriechbaumer V, Müller A, Piotrowski M, Meeley RB, Gierl A, Glawischnig E. The nitrilase ZmNIT2 converts indole-3-acetonitrile to indole-3-acetic acid. Plant Physiol. 2003;133(2):794–802. 12972653 10.1104/pp.103.026609 PMC219053

[118] Braybrook SA, Harada JJ. LECs go crazy in embryo development. Trends Plant Sci. 2008;13(12):624–30. 19010711 10.1016/j.tplants.2008.09.008

[119] Chow CN, Lee TY, Hung YC, Li GZ, Tseng KC, Liu YH, Kuo PL, Zheng HQ, Chang WC. PlantPAN3.0: a new and updated resource for reconstructing transcriptional regulatory networks from ChIP-seq experiments in plants. Nucleic Acids Res. 2019;47(D1):D1155–63. 30395277 10.1093/nar/gky1081 PMC6323957

[120] Kutz A, Müller A, Hennig P, Kaiser WM, Piotrowski M, Weiler EW. A role for nitrilase 3 in the regulation of root morphology in sulphur-starving *Arabidopsis thaliana*. Plant J. 2002;30(1):95–106. 11967096 10.1046/j.1365-313X.2002.01271.x

[121] van der Woude L, Piotrowski M, Klaasse G, Paulus JK, Krahn D, Ninck S, Kaschani F, Kaiser M, Novák O, Ljung K. The chemical compound ‘Heatin’stimulates hypocotyl elongation and interferes with the Arabidopsis NIT1-subfamily of nitrilases. Plant J. 2021;106(6):1523–40. 33768644 10.1111/tpj.15250 PMC8360157

[122] Zavattieri MA, Frederico AM, Lima M, Sabino R, Arnholdt-Schmitt B. Induction of somatic embryogenesis as an example of stress-related plant reactions. Electron J Biotechnol. 2010;13(1):12–3. 10.2225/vol13-issue1-fulltext-4

[123] Kasahara H. Current aspects of auxin biosynthesis in plants. Biosci Biotechnol Biochem. 2016;80(1):34–42. 26364770 10.1080/09168451.2015.1086259

[124] Machingura M, Salomon E, Jez JM, Ebbs SD. The beta-cyanoalanine synthase pathway: beyond cyanide detoxification. Plant Cell Environ. 2016;39(10):2329–41. 27116378 10.1111/pce.12755

[125] Su YH, Zhao XY, Liu YB, Zhang CL, O’Neill SD, Zhang XS. Auxin-induced *WUS* expression is essential for embryonic stem cell renewal during somatic embryogenesis in Arabidopsis. Plant J. 2009;59(3):448–60. 19453451 10.1111/j.1365-313X.2009.03880.x PMC2788036

[126] Jia Y, Tian H, Li H, Yu Q, Wang L, Friml J, Ding Z. The Arabidopsis thaliana Elongator complex subunit 2 epigenetically affects root development. J Exp Bot. 2015;66(15):4631–42. 25998905 10.1093/jxb/erv230 PMC4507768

[127] Tanaka H, Dhonukshe P, Brewer PB, Friml J. Spatiotemporal asymmetric auxin distribution: a means to coordinate plant development. Cell Mol Life Sci. 2006;63(23):2738–54. 17013565 10.1007/s00018-006-6116-5 PMC11136431

[128] Song S, Wang Z, Ren Y, Sun H. Full-length transcriptome analysis of the *ABCB*, *PIN/PIN-LIKE*S, and *AUX/LAX* families involved in somatic embryogenesis of *Lilium pumilum* DC. Fisch. Int J Mol Sci. 2020;21(2):453. 31936841 10.3390/ijms21020453 PMC7014436

[129] Elhiti M, Stasolla C. Transduction of signals during somatic embryogenesis. Plants (Basel). 2022;11(2):178. 35050066 10.3390/plants11020178 PMC8779037

[130] Dai L, He G, Zhang K, Guan X, Wang Y, Zhang B. Trichostatin A induces p53-dependent endoplasmic reticulum stress in human colon cancer cells. Oncol Lett. 2019;17(1):660–7. 30655814 10.3892/ol.2018.9641 PMC6313176

[131] Chen LT, Luo M, Wang YY, Wu K. Involvement of Arabidopsis histone deacetylase HDA6 in ABA and salt stress response. J Exp Bot. 2010;61(12):3345–53. 20519338 10.1093/jxb/erq154 PMC2905197

[132] Zheng Y, Ding Y, Sun X, Xie S, Wang D, Liu X, Su L, Wei W, Pan L, Zhou DX. Histone deacetylase HDA9 negatively regulates salt and drought stress responsiveness in Arabidopsis. J Exp Bot. 2016;67(6):1703–13. 26733691 10.1093/jxb/erv562

[133] Feng C, Cai XW, Su YN, Li L, Chen S, He XJ. Arabidopsis RPD3-like histone deacetylases form multiple complexes involved in stress response. J Genet Genomics. 2021;48(5):369–83. 34144927 10.1016/j.jgg.2021.04.004

[134] Jadko S. Histone deacetylase activity and reactive oxygen species content in the tissue culture of *Arabidopsis thaliana* under normal conditions and development of acute osmotic stress. Ukr Biochem J 2015(87,№ 3):57–62. 26502700

[135] Kudełko K, Gaj MD. Glutathione (GSH) induces embryogenic response in *in vitro* cultured explants of *Arabidopsis thaliana* via auxin-related mechanism. Plant Growth Regul. 2019;89(1):25–36. 10.1007/s10725-019-00514-1

[136] Herrera-Vásquez A, Salinas P, Holuigue L. Salicylic acid and reactive oxygen species interplay in the transcriptional control of defense genes expression. Front Plant Sci. 2015;6:171. 25852720 10.3389/fpls.2015.00171 PMC4365548

[137] Nazar R, Umar S, Khan NA. Exogenous salicylic acid improves photosynthesis and growth through increase in ascorbate-glutathione metabolism and S assimilation in mustard under salt stress. Plant Signal Behav. 2015;10(3):e1003751. 25730495 10.1080/15592324.2014.1003751 PMC4622964

[138] Su YH, Su YX, Liu YG, Zhang XS. Abscisic acid is required for somatic embryo initiation through mediating spatial auxin response in Arabidopsis. Plant Growth Regul. 2012;69(2):167–76. 10.1007/s10725-012-9759-2

[139] Chen Y, Yu H, Wang Y, Li F, Xing Y, Ge X. Uniconazole augments Abscisic Acid in promoting somatic embryogenesis in cotton (Gossypium hirsutum L). Front Plant Sci. 2022;13:865778. 35444669 10.3389/fpls.2022.865778 PMC9014122

[140] Choi SM, Song HR, Han SK, Han M, Kim CY, Park J, Lee YH, Jeon JS, Noh YS, Noh B. HDA19 is required for the repression of salicylic acid biosynthesis and salicylic acid-mediated defense responses in Arabidopsis. Plant J. 2012;71(1):135–46. 22381007 10.1111/j.1365-313X.2012.04977.x

[141] Wu Z, He L, Jin Y, Chen J, Shi H, Wang Y, Yang W. HISTONE DEACETYLASE 6 suppresses salicylic acid biosynthesis to repress autoimmunity. Plant Physiol. 2021;187(4):2592–607. 34618093 10.1093/plphys/kiab408 PMC8644357

[142] Chen J, Clinton M, Qi G, Wang D, Liu F, Fu ZQ. Reprogramming and remodeling: transcriptional and epigenetic regulation of salicylic acid-mediated plant defense. J Exp Bot. 2020;71(17):5256–68. 32060527 10.1093/jxb/eraa072

[143] Gu D, Yang J, Wu S, Liao Y, Zeng L, Yang Z. Epigenetic regulation of the phytohormone abscisic acid accumulation under dehydration stress during postharvest processing of tea (*Camellia sinensis*). J Agric Food Chem. 2021;69(3):1039–48. 33464046 10.1021/acs.jafc.0c07220

[144] Wang Z, Cao H, Sun Y, Li X, Chen F, Carles A, Li Y, Ding M, Zhang C, Deng X, et al. Arabidopsis paired amphipathic helix proteins SNL1 and SNL2 redundantly regulate primary seed dormancy via abscisic acid-ethylene antagonism mediated by histone deacetylation. Plant Cell. 2013;25(1):149–66. 23371947 10.1105/tpc.112.108191 PMC3584531

[145] Truong HA, Lee S, Trinh CS, Lee WJ, Chung EH, Hong SW, Lee H. Overexpression of the *HDA15* gene confers resistance to salt stress by the induction of NCED3, an ABA biosynthesis enzyme. Front Plant Sci. 2021;12:640443. 33995439 10.3389/fpls.2021.640443 PMC8120240

[146] Mira MM, Wally OS, Elhiti M, El-Shanshory A, Reddy DS, Hill RD, Stasolla C. Jasmonic acid is a downstream component in the modulation of somatic embryogenesis by Arabidopsis Class 2 phytoglobin. J Exp Bot. 2016;67(8):2231–46. 26962208 10.1093/jxb/erw022 PMC4809281

[147] Karami O, Saidi A. The molecular basis for stress-induced acquisition of somatic embryogenesis. Mol Biol Rep. 2010;37(5):2493–507. 19705297 10.1007/s11033-009-9764-3

[148] Lai C, Zhou X, Zhang S, Zhang X, Liu M, Zhang C, Xu X, Xu X, Chen X, Chen Y, et al. PAs regulate early somatic embryo development by changing the gene expression level and the hormonal balance in *Dimocarpus longan* lour. Genes (Basel). 2022;13(2):317. 35205362 10.3390/genes13020317 PMC8872317

[149] Wu YJ, Huang XL, Chen QZ, Li XJ, Engelmann F. Induction and cryopreservation of embryogenic cultures from nucelli and immature cotyledon cuts of mango (Mangifera indica L. var Zihua). Plant Cell Rep. 2007;26(2):161–8. 16972094 10.1007/s00299-006-0232-4

[150] Chiappetta A, Fambrini M, Petrarulo M, Rapparini F, Michelotti V, Bruno L, Greco M, Baraldi R, Salvini M, Pugliesi C, et al. Ectopic expression of LEAFY COTYLEDON1-LIKE gene and localized auxin accumulation mark embryogenic competence in epiphyllous plants of Helianthus annuus x H. tuberosus. Ann Bot. 2009;103(5):735–47. 19151043 10.1093/aob/mcn266 PMC2707873

[151] Eshed Y, Baum SF, Perea JV, Bowman JL. Establishment of polarity in lateral organs of plants. Curr Biol. 2001;11(16):1251–60. 11525739 10.1016/S0960-9822(01)00392-X

[152] Emery JF, Floyd SK, Alvarez J, Eshed Y, Hawker NP, Izhaki A, Baum SF, Bowman JL. Radial patterning of Arabidopsis shoots by class III HD-ZIP and KANADI genes. Curr Biol. 2003;13(20):1768–74. 14561401 10.1016/j.cub.2003.09.035

[153] Kurczyńska EU, Gaj MD, Ujczak A, Mazur E. Histological analysis of direct somatic embryogenesis in Arabidopsis thaliana (L.) Heynh. Planta. 2007;226(3):619–28. 17406890 10.1007/s00425-007-0510-6

[154] Burian A, Paszkiewicz G, Nguyen KT, Meda S, Raczyńska-Szajgin M, Timmermans MCP. Specification of leaf dorsiventrality via a prepatterned binary readout of a uniform auxin input. Nat Plants. 2022;8(3):269–80. 35318449 10.1038/s41477-022-01111-3

[155] Du F, Guan C, Jiao Y. Molecular mechanisms of leaf morphogenesis. Mol Plant. 2018;11(9):1117–34. 29960106 10.1016/j.molp.2018.06.006

[156] Magnani E, Jiménez-Gómez JM, Soubigou-Taconnat L, Lepiniec L, Fiume E. Profiling the onset of somatic embryogenesis in Arabidopsis. BMC Genom. 2017;18(1):998. 10.1186/s12864-017-4391-1 PMC5747089 29284399

[157] Wang FX, Shang GD, Wu LY, Xu ZG, Zhao XY, Wang JW. Chromatin accessibility dynamics and a hierarchical transcriptional regulatory network structure for plant somatic embryogenesis. Dev Cell. 2020;54(6):742–57. 32755547 10.1016/j.devcel.2020.07.003

[158] Guan C, Wu B, Yu T, Wang Q, Krogan NT, Liu X, Jiao Y. Spatial auxin signaling controls leaf flattening in Arabidopsis. Curr Biol. 2017;27(19):2940–50. 28943086 10.1016/j.cub.2017.08.042 PMC6419953

[159] Heisler MG, Byrne ME. Progress in understanding the role of auxin in lateral organ development in plants. Curr Opin Plant Biol. 2020;53:73–9. 31785585 10.1016/j.pbi.2019.10.007

[160] Horstman A, Willemsen V, Boutilier K, Heidstra R. AINTEGUMENTA-LIKE proteins: hubs in a plethora of networks. Trends Plant Sci. 2014;19(3):146–57. 24280109 10.1016/j.tplants.2013.10.010

[161] Dong X, Yan Y, Jiang B, Shi Y, Jia Y, Cheng J, Shi Y, Kang J, Li H, Zhang D, et al. The cold response regulator CBF1 promotes Arabidopsis hypocotyl growth at ambient temperatures. EMBO J. 2020;39(13):e103630. 32449547 10.15252/embj.2019103630 PMC7327500

[162] Sun J, Qi L, Li Y, Chu J, Li C. PIF4-mediated activation of *YUCCA8* expression integrates temperature into the auxin pathway in regulating Arabidopsis hypocotyl growth. PLoS Genet. 2012;8(3):e1002594. 22479194 10.1371/journal.pgen.1002594 PMC3315464

[163] Chinnusamy V, Ohta M, Kanrar S, Lee BH, Hong X, Agarwal M, Zhu JK. ICE1: a regulator of cold-induced transcriptome and freezing tolerance in Arabidopsis. Genes Dev. 2003;17(8):1043–54. 12672693 10.1101/gad.1077503 PMC196034

[164] Le BH, Cheng C, Bui AQ, Wagmaister JA, Henry KF, Pelletier J, Kwong L, Belmonte M, Kirkbride R, Horvath S, et al. Global analysis of gene activity during Arabidopsis seed development and identification of seed-specific transcription factors. Proc Natl Acad Sci U S A. 2010;107(18):8063–70. 20385809 10.1073/pnas.1003530107 PMC2889569

[165] Carles CC, Choffnes-Inada D, Reville K, Lertpiriyapong K, Fletcher JC. *ULTRAPETALA1* encodes a SAND domain putative transcriptional regulator that controls shoot and floral meristem activity in Arabidopsis. Development. 2005;132(5):897–911. 15673576 10.1242/dev.01642

[166] Ornelas-Ayala D, Vega-León R, Petrone-Mendoza E, Garay-Arroyo A, García-Ponce B, Álvarez-Buylla ER, Sanchez MP. *ULTRAPETALA1* maintains Arabidopsis root stem cell niche independently of *ARABIDOPSIS TRITHORAX1*. New Phytol. 2020;225(3):1261–72. 31545512 10.1111/nph.16213

[167] Zuo J, Niu QW, Frugis G, Chua NH. The *WUSCHEL* gene promotes vegetative-to‐embryonic transition in Arabidopsis. Plant J. 2002;30(3):349–59. 12000682 10.1046/j.1365-313X.2002.01289.x

[168] Carles CC, Lertpiriyapong K, Reville K, Fletcher JC. The *ULTRAPETALA1* gene functions early in Arabidopsis development to restrict shoot apical meristem activity and acts through *WUSCHEL* to regulate floral meristem determinacy. Genetics. 2004;167(4):1893–903. 15342527 10.1534/genetics.104.028787 PMC1471006

[169] Wang Y-B, Song J-P, Wang Z-B. *ASYMMETRIC LEAVES2-LIKE38*, one member of AS2/LOB gene family, involves in regulating ab-adaxial patterning in Arabidopsis lateral organs. *Acta Physiol Plant* 2015, 37(9):185.

[170] Kang NY, Lee HW, Kim J. The AP2/EREBP gene PUCHI co-acts with LBD16/ASL18 and LBD18/ASL20 downstream of ARF7 and ARF19 to regulate lateral root development in Arabidopsis. Plant Cell Physiol. 2013;54(8):1326–34. 23749813 10.1093/pcp/pct081

[171] Sugimoto K, Jiao Y, Meyerowitz EM. Arabidopsis regeneration from multiple tissues occurs via a root development pathway. Dev Cell. 2010;18(3):463–71. 20230752 10.1016/j.devcel.2010.02.004

[172] Tang LP, Zhou C, Wang SS, Yuan J, Zhang XS, Su YH. FUSCA3 interacting with LEAFY COTYLEDON2 controls lateral root formation through regulating *YUCCA4* gene expression in *Arabidopsis thaliana*. New Phytol. 2017;213(4):1740–54. 27878992 10.1111/nph.14313

[173] Pandey SK, Lee HW, Kim MJ, Cho C, Oh E, Kim J. LBD18 uses a dual mode of a positive feedback loop to regulate ARF expression and transcriptional activity in Arabidopsis. Plant J. 2018;95(2):233–51. 29681137 10.1111/tpj.13945

[174] Ueno Y, Ishikawa T, Watanabe K, Terakura S, Iwakawa H, Okada K, Machida C, Machida Y. Histone deacetylases and ASYMMETRIC LEAVES2 are involved in the establishment of polarity in leaves of Arabidopsis. Plant Cell. 2007;19(2):445–57. 17293570 10.1105/tpc.106.042325 PMC1867339

