# Supplementary figures and images for: Transcriptomic profiling reveals histone acetylation-regulated genes involved in somatic embryogenesis in Arabidopsis thaliana
Source: BMC Genomics. 2024 Aug 15;25:788. doi: 10.1186/s12864-024-10623-5 (PMC11325840; doi:10.1186/s12864-024-10623-5)

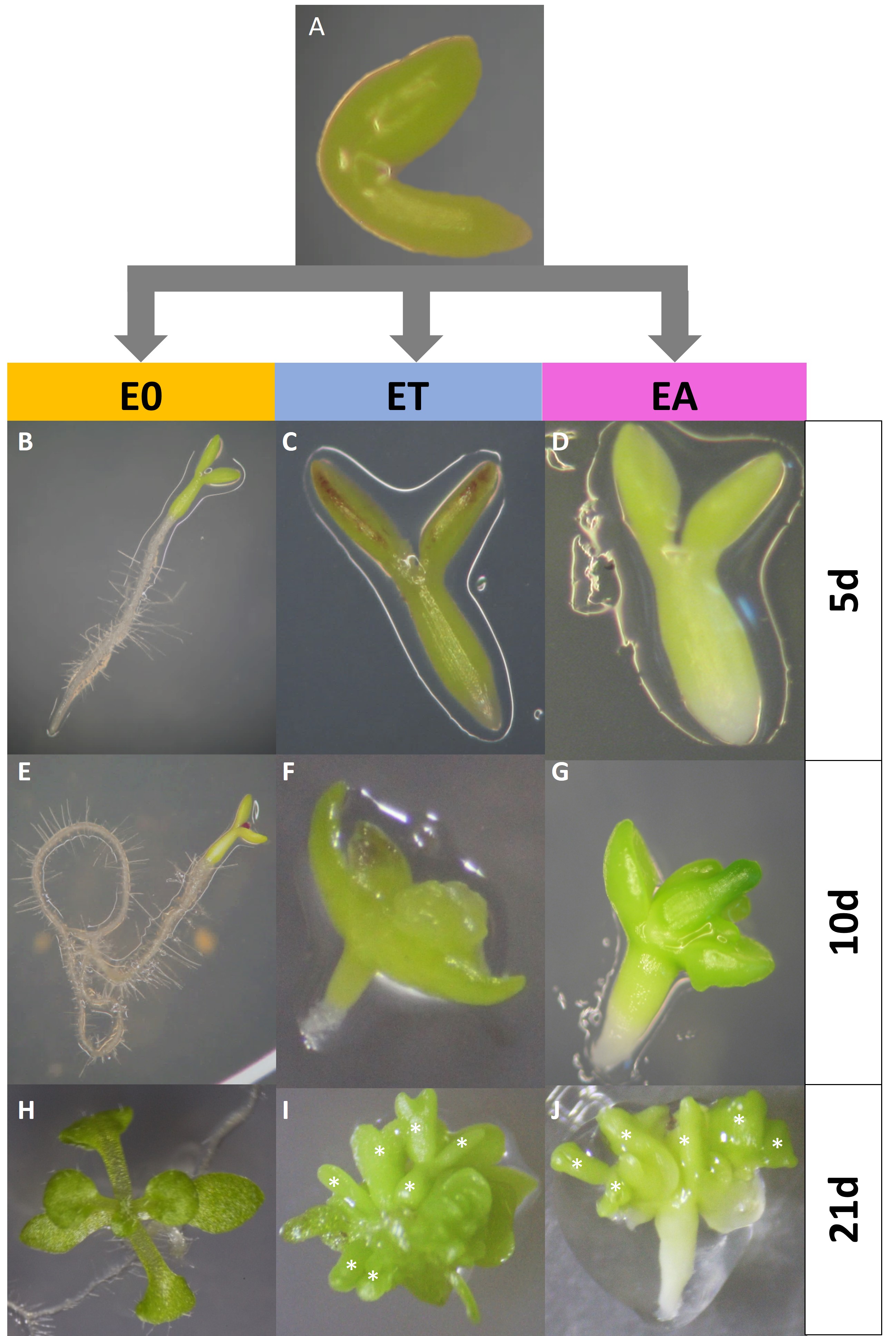

Supplement: Supplementary file 2 — Additional file 2: Fig. S1 [file 12864_2024_10623_MOESM2_ESM.jpg]

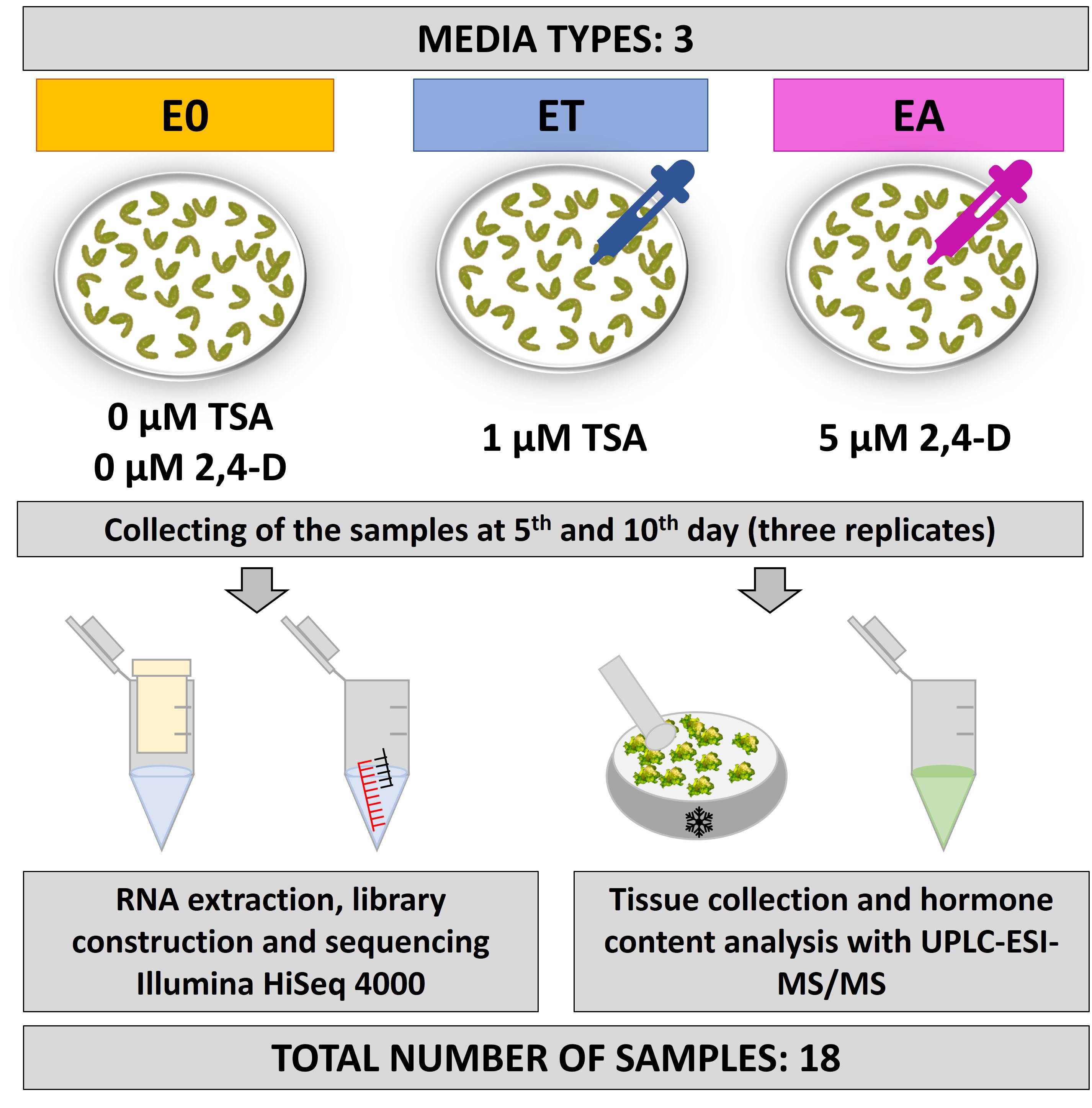

Supplement: Supplementary file 3 — Additional file 3: Fig. S2 [file 12864_2024_10623_MOESM3_ESM.jpg]

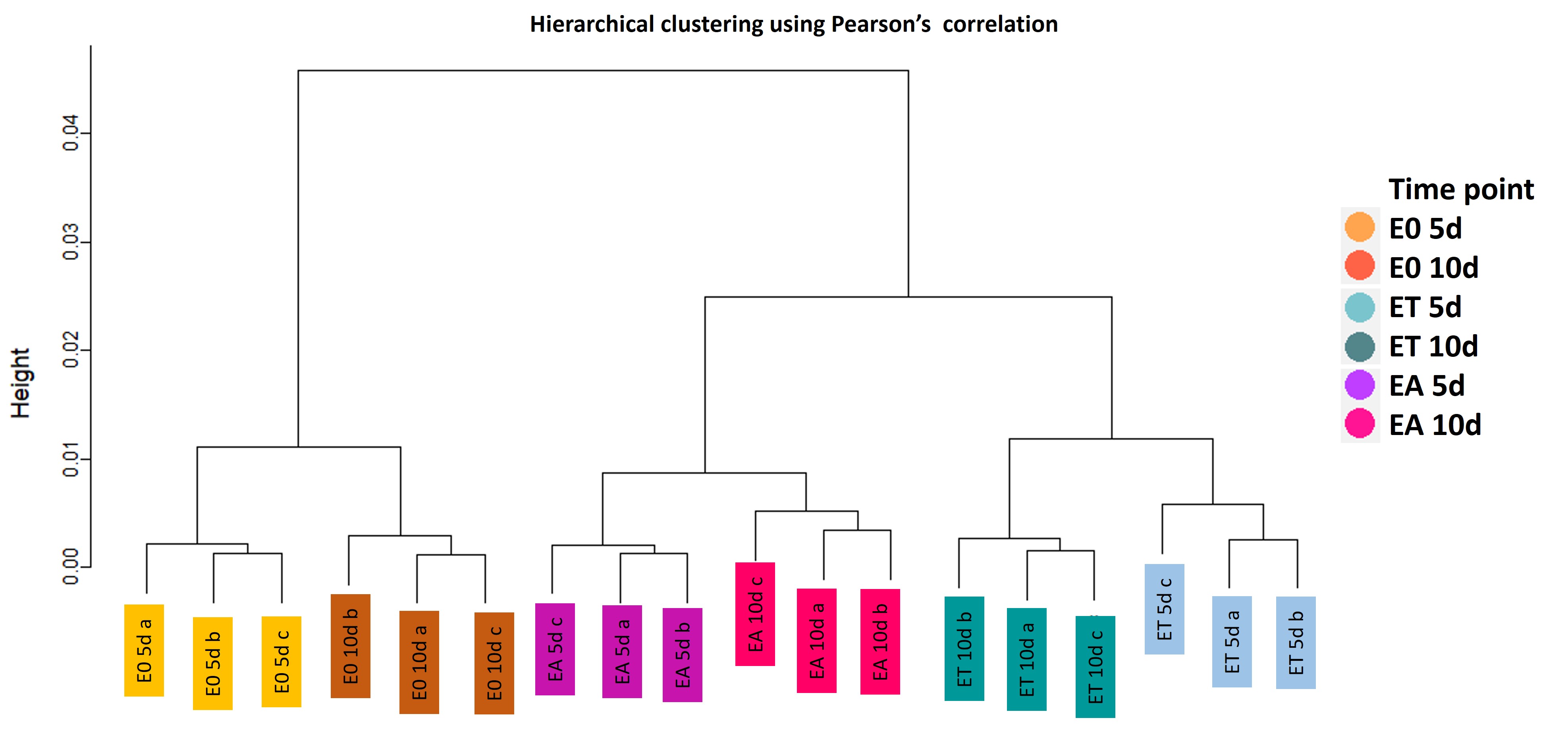

Supplement: Supplementary file 4 — Additional file 4: Fig. S3 [file 12864_2024_10623_MOESM4_ESM.jpg]

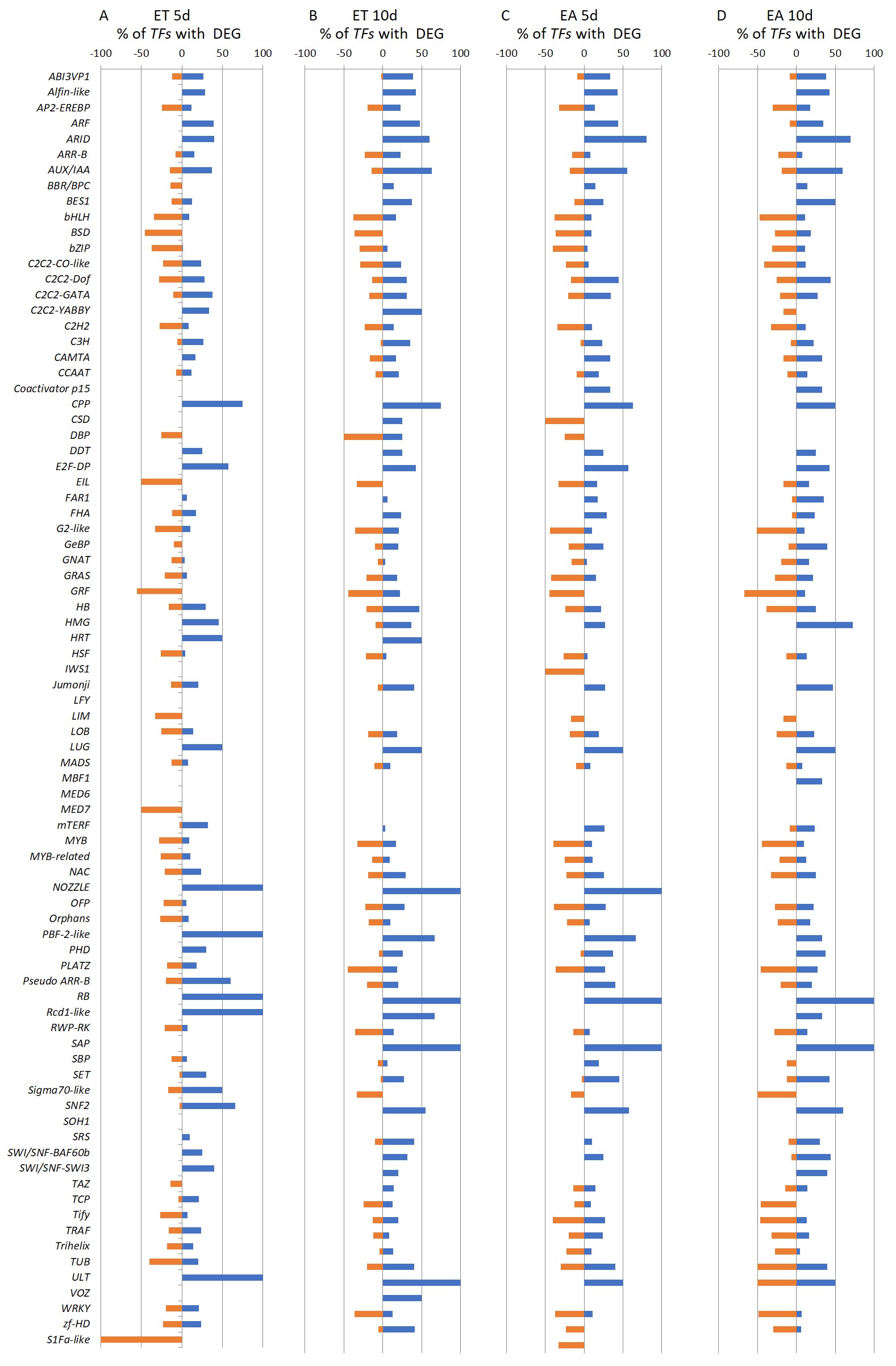

Supplement: Supplementary file 8 — Additional file 8: Fig. S4 [file 12864_2024_10623_MOESM8_ESM.jpg]

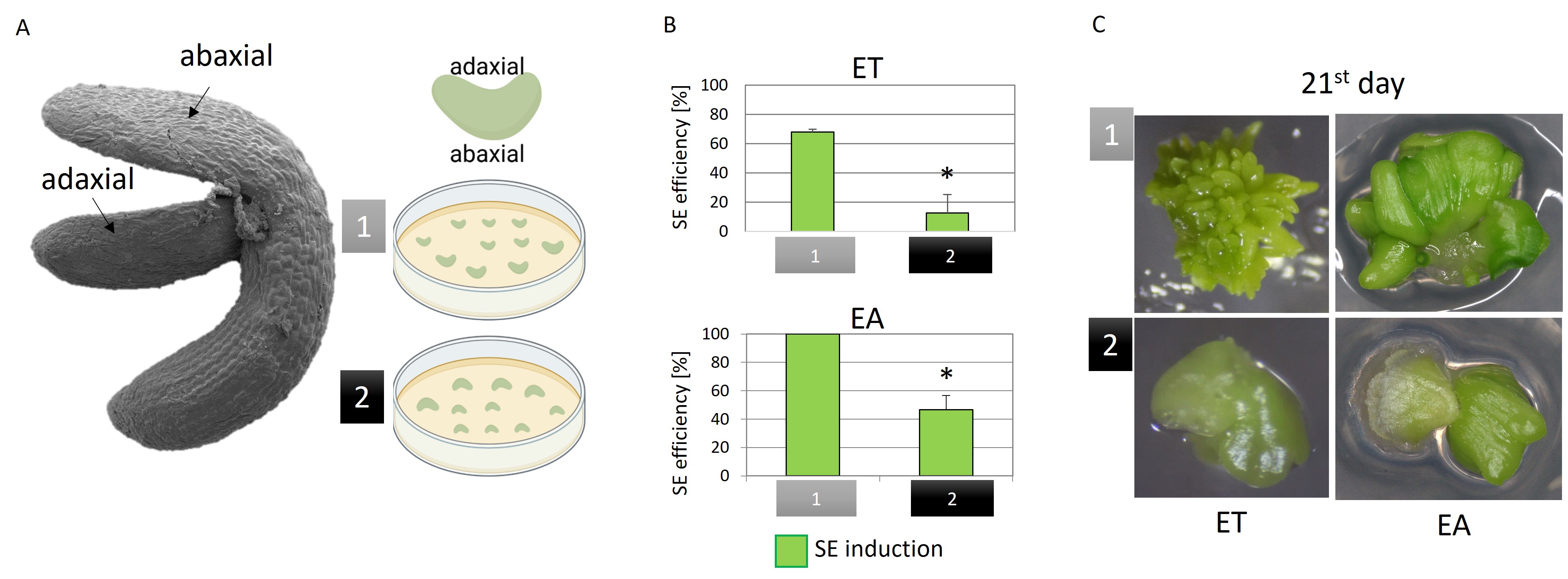

Supplement: Supplementary file 15 — Additional file 15: Fig. S5 [file 12864_2024_10623_MOESM15_ESM.jpg]

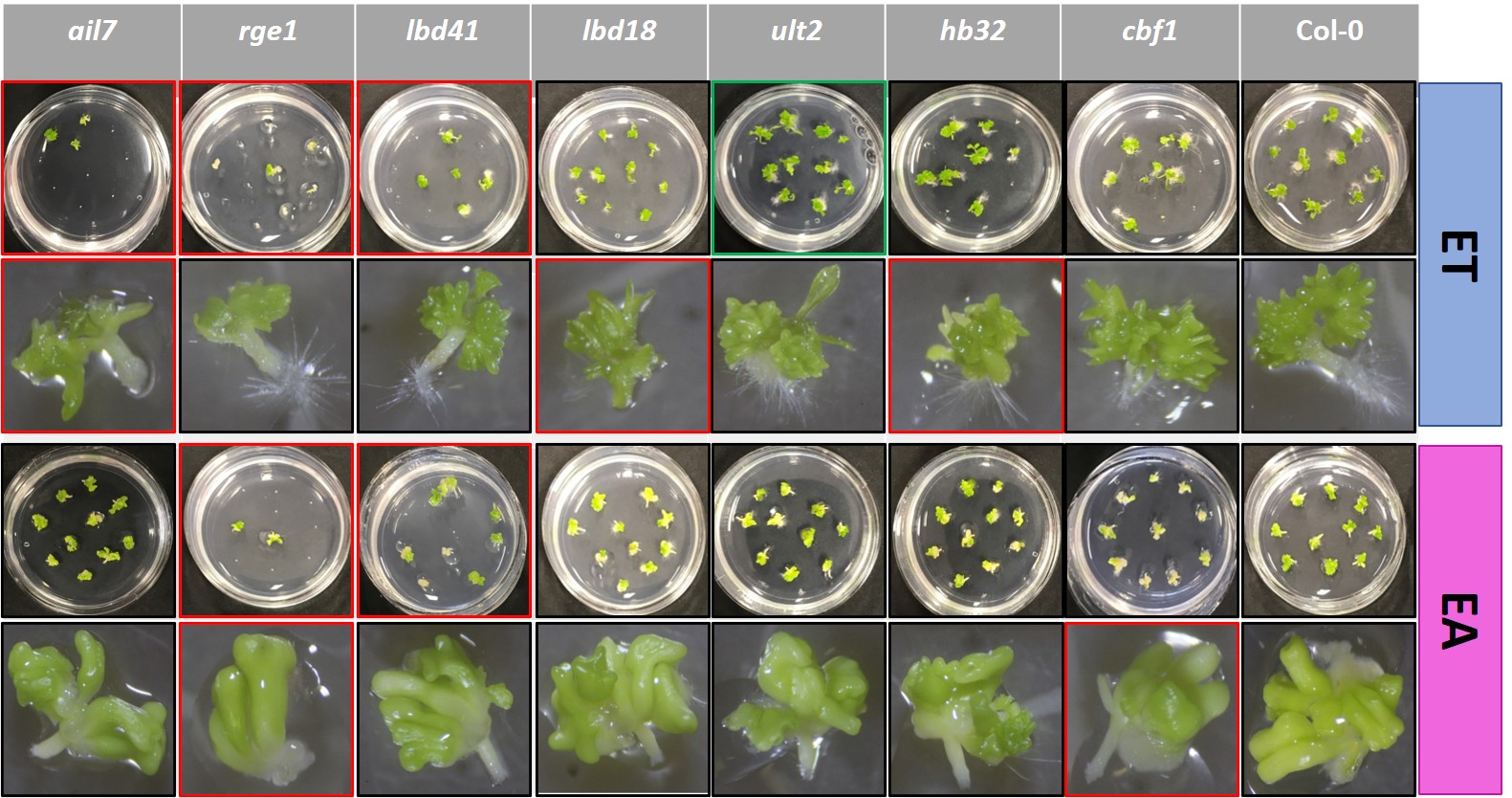

Supplement: Supplementary file 17 — Additional file 17: Fig. S6 [file 12864_2024_10623_MOESM17_ESM.jpg]
