# Supplementary material for: Transcriptomic profiling reveals histone acetylation-regulated genes involved in somatic embryogenesis in Arabidopsis thaliana
Source: BMC Genomics. 2024 Aug 15;25:788. doi: 10.1186/s12864-024-10623-5 (PMC11325840; doi:10.1186/s12864-024-10623-5)
Supplement: Supplementary file 18 — Additional file 18: Legends [file 12864_2024_10623_MOESM18_ESM.docx]

**Additional files:**

**Additional file1** Table S1. The insertional mutants and the primers used in experiments.

**
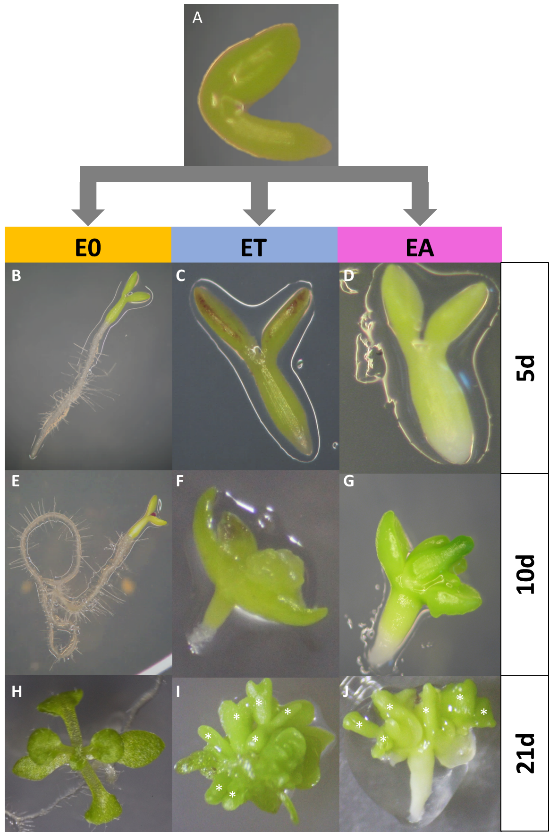
**

**Additional file2** Figure S1. *In vitro* induced embryogenic response of Arabidopsis (Col-0) explants, immature zygotic embryo explants (IZEs), to HDAC inhibitor (TSA) and auxin (2,4-D); E0 control medium free of TSA and auxin (B, E, H); ET medium with TSA (C, F, I), EA medium with auxin (D, G, J). Explants at 0 (A), 5^th^ (B, C, D), 10^th^ (E, F, G), and 21^st^ day (H, I, J) of *in vitro* culture. Seedling development (B, E, H). Embryogenic responses: explant enlargement (C, D); tissue proliferation and somatic embryo-like protuberances at the adaxial side of IZE cotyledons (F, G); somatic embryos (I, J) marked with asterisks.


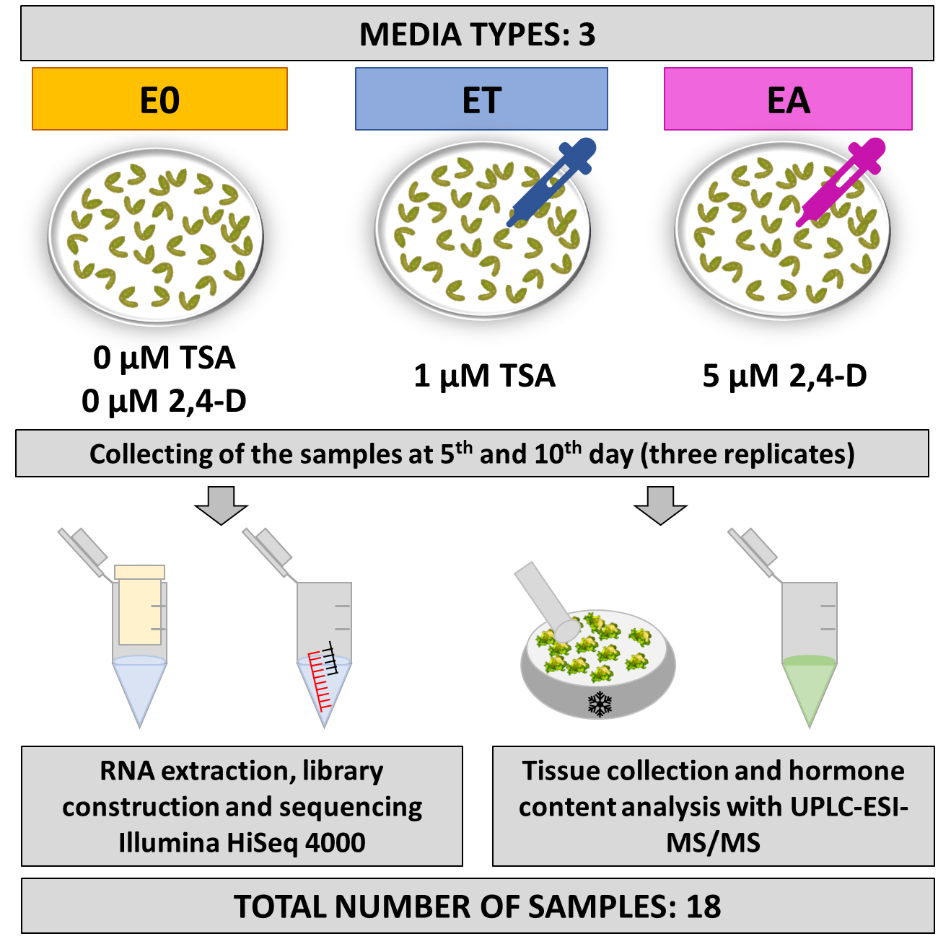


**Additional file3** Figure S2. Graphical illustration of the experimental design for RNA-seq analysis. IZE explants of Arabidopsis (Col-0) were cultured on three media – non-embryogenic E0 and SE-inducing supplemented with 1 µM TSA (ET) and 5 µM 2,4-D (EA). Explants on the 5^th^ and 10^th^ day of *in vitro* culture were collected for transcriptome sequencing and analysis of hormone content.


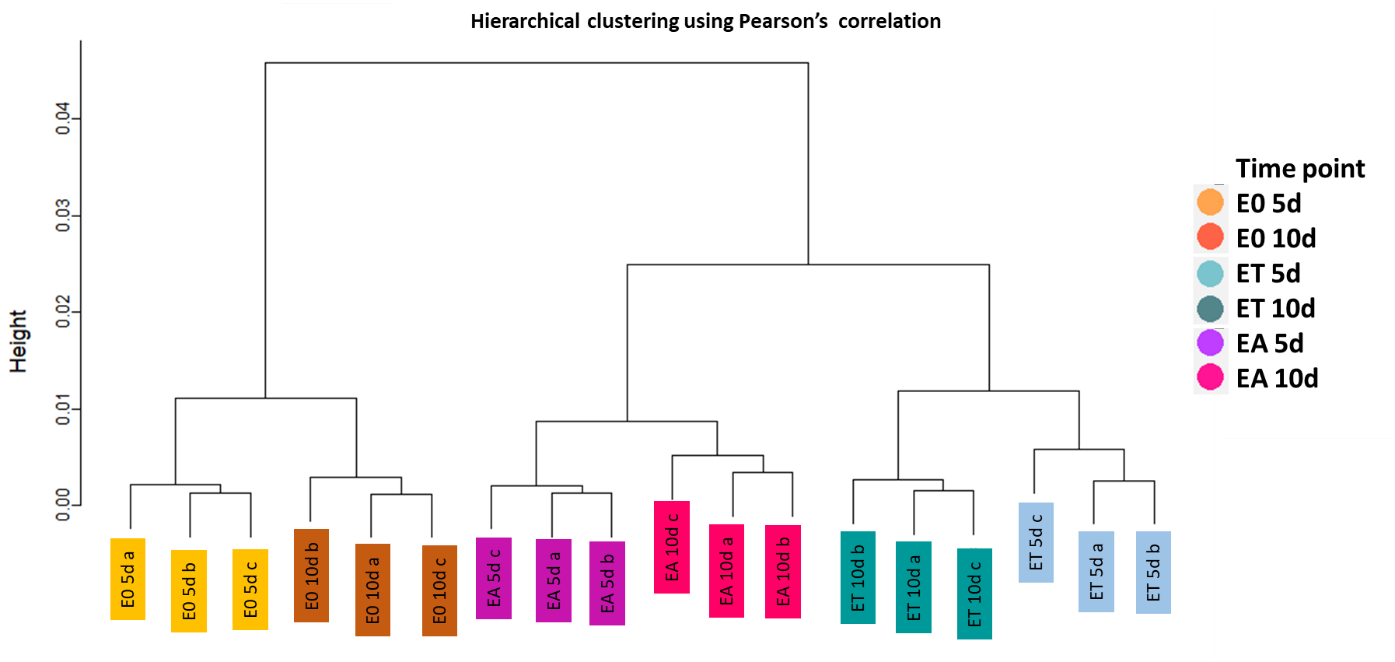


**Additional file4** Figure S3. Hierarchical clustering of mRNA-seq libraries of explants cultured *in vitro* for 5 and 10 days on control (E0) and SE-induced media with TSA (ET) and auxin (EA). The dendrogram was produced by clustering Pearson’s correlation of log_2_ (TPM + 1) using Ward’s criterion; a, b, c - biological replicates.

**Additional file5** Dataset S1. DEGs in the embryogenic culture of explants induced on TSA (ET) and auxin (EA) medium in relevance to non-embryogenic E0 culture. Values represent the relative expression level (log fold change, logFC) in the ET vs. E0 and EA vs. E0 media on the 5^th^ and 10^th^ day of explant culture. Wald's exact test was used to identify any differentially expressed genes (DEGs) under a p-value adjustment (p < 0.05) for multiple comparisons with the Benjamini-Hochberg False Discovery Rate (FDR) correction.

**Additional file6** Dataset S2. GO analysis of DEGs of ET (TSA)- and auxin (EA)-specific expression.

**Additional file7** Dataset S3. GO analysis of up- and down-regulated DEGs in TSA (ET) and EA (auxin)-induced SE at 5^th^ and 10^th^ day of culture.


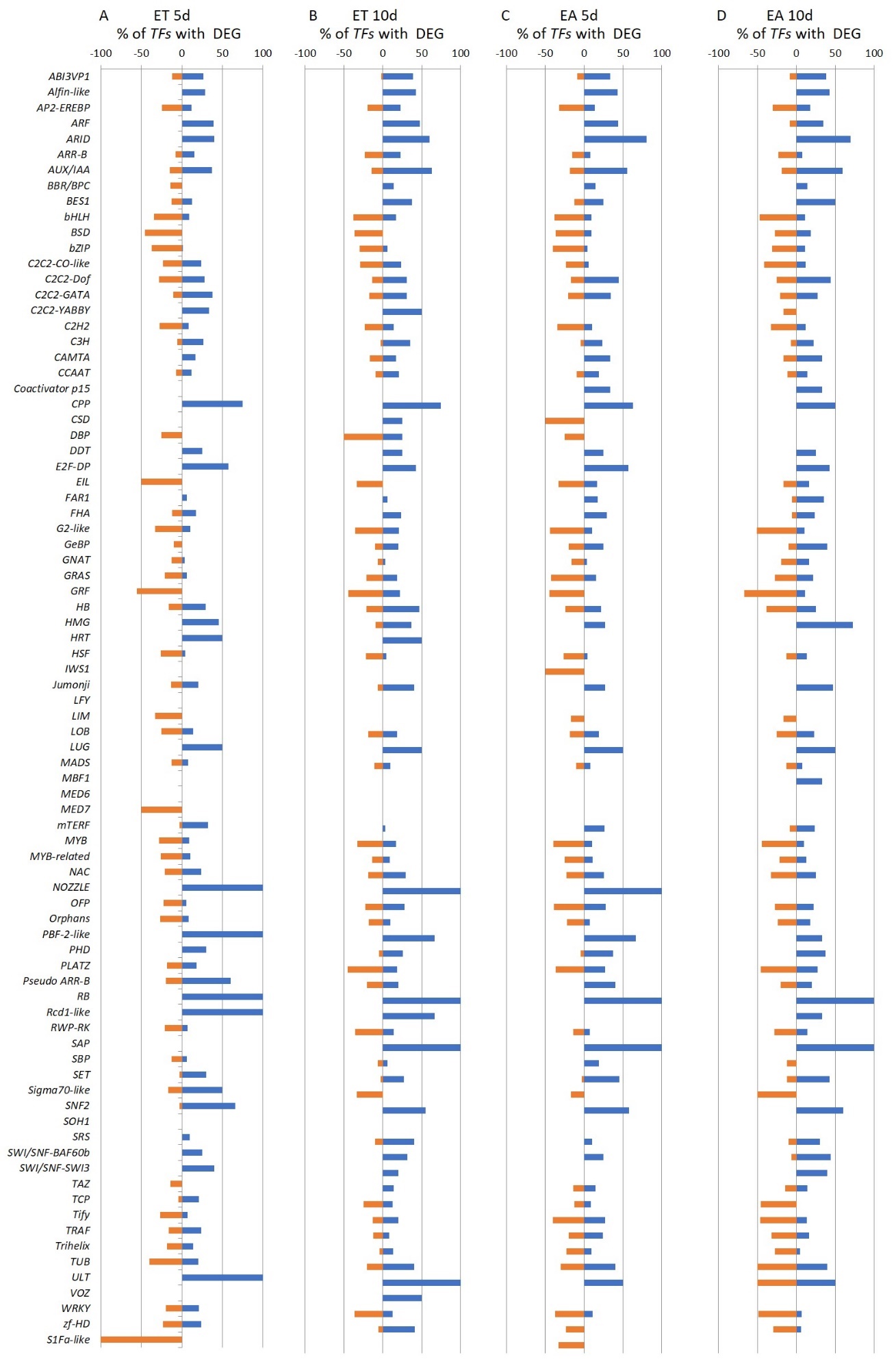


**Additional file8** Figure S4. Percentage of differentially expressed (DEGs) *TFs* from different gene families during SE induction on ET (A, B) or EA (C, D) medium at 5^th^ (A, C) and 10^th^ (B, D) day, respectively.

**Additional file9** Dataset S4. Differentially expressed *TF* genes in TSA (ET)- and auxin (EA)-induced embryogenic cultures

**Additional file10** Table S2. Expression levels of the SE-regulator genes in EA- and ET-induced explant cultures. Values represent the relative expression level (fold change, FC) in the ET vs. E0 and EA vs. E0 cultures on the 5^th^ and 10^th^ day. Wald's exact test was used to identify any differentially expressed genes (DEGs) under a p-value adjustment (p < 0.05) for multiple comparisons with the Benjamini-Hochberg False Discovery Rate (FDR) correction.

**Additional file11** Dataset S5. Differentially expressed genes in TSA-induced explants in relevance to auxin-induced culture, DEGs-ET/EA. Values represent the relative expression level (log fold change, logFC) in the ET vs. EA culture on the 5^th^ and 10^th^ day. Wald's exact test was used to identify any differentially expressed genes (DEGs) under a p-value adjustment (p < 0.05) for multiple comparisons with the Benjamini-Hochberg False Discovery Rate (FDR) correction.

**Additional file12** Dataset S6. GO analysis of genes differentially expressed in ET compared to EA culture (DEGs-ET/EA) on the 5^th^ and 10^th^ day of SE induction.

**Additional file13** Table S3. DEGs related to auxin metabolism, transport, and signaling. Values represent the relative gene expression level (log2FC) in the ET vs. E0, EA vs. E0, and ET vs. EA culture on the 5^th^ and 10^th^ day. Wald's exact test was used to identify any differentially expressed genes (DEGs) under a p-value adjustment (p < 0.05) for multiple comparisons with the Benjamini-Hochberg False Discovery Rate (FDR) correction.

**Additional file14** Table S4. The expression level of genes related to SA, ABA, and JA biosynthesis in the embryogenic culture of explants induced on TSA (ET) and auxin (EA) medium in relevance to non-embryogenic E0 culture on 5^th^ and 10^th^ day. Values represent the relative expression level (log2FC) in the ET vs. E0, EA vs. E0, and ET vs. EA medium on 5^th^ and 10^th^ day of culture. Wald's exact test was used to identify any differentially expressed genes (DEGs) under a p-value adjustment (p < 0.05) for multiple comparisons with the Benjamini-Hochberg False Discovery Rate (FDR) correction.


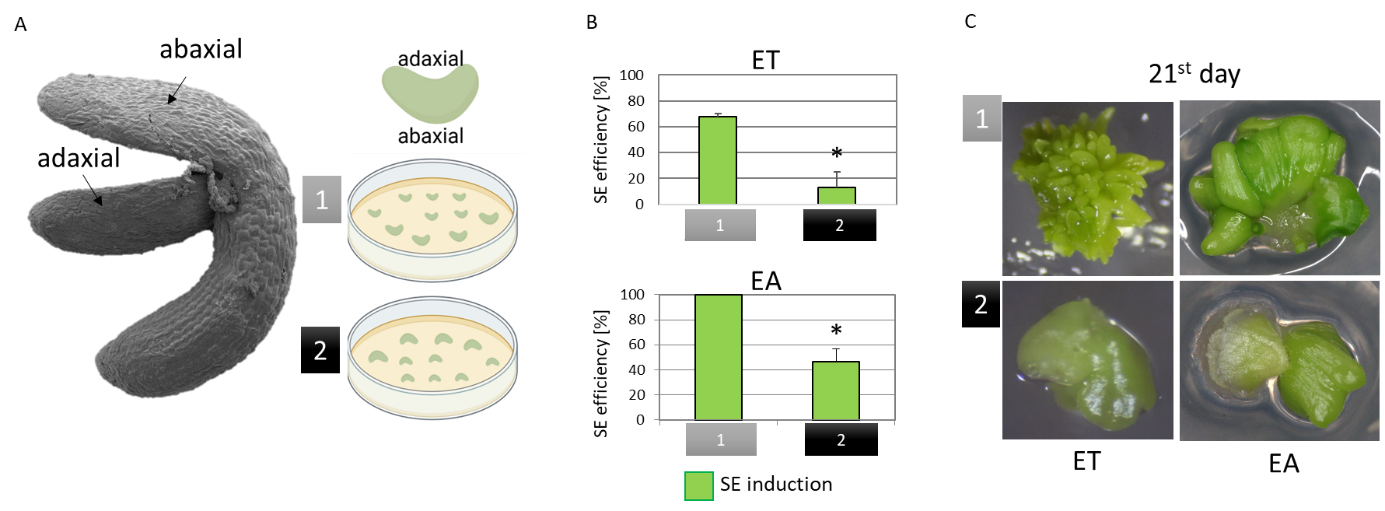


**Additional file15** Figure S5. The impact of explant polarity on somatic embryogenesis induction. The graphical illustration of the experiment: cotyledons isolated from Col-0 immature zygotic embryos were oriented with the adaxial (1) or abaxial (2) side up to the SE-induction medium (A). SE efficiency (B) of the adaxial- and abaxial side-oriented on the ET and EA medium explants. Values significantly different from the abaxial-side-oriented explants are marked with an asterisk (n = 3; means ± SD are given) (Student’s t-test, p < 0.05). The high SE induction from the adaxial side of cotyledon explants (C).

**Additional file16** Table S5. The expression level of the organ polarity-related in the embryogenic culture of explants induced on TSA (ET) and auxin (EA) medium in relevance to non-embryogenic E0 culture on 5^th^ and 10^th^ day. Data from the RNA-seq analysis are given. Wald's exact test was used to identify any differentially expressed genes (DEGs) under a p-value adjustment (p < 0.05) for multiple comparisons with the Benjamini-Hochberg False Discovery Rate (FDR) correction.


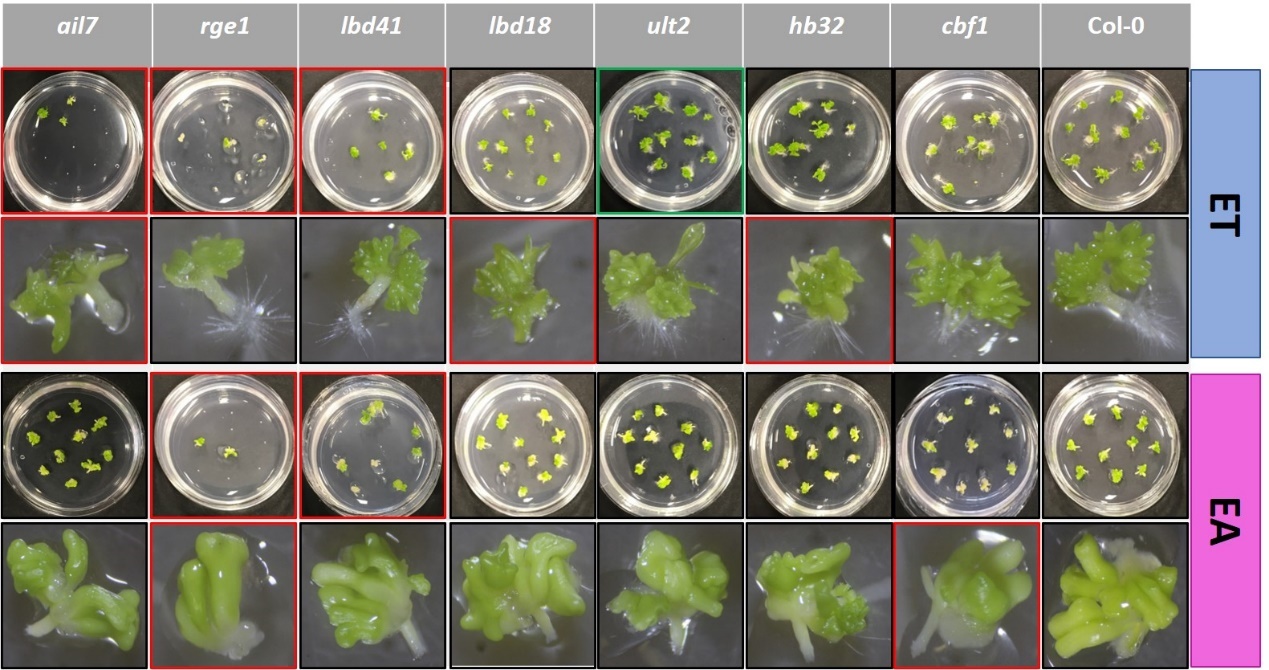


**Additional file17** Figure S6. The embryogenic response of the polarity-related mutants (*ail7, rge1, lbd41, lbd18, ult2, hb32, cbf1*) and WT (Col-0) genotype on the ET and EA media on 21^st^ day.
